# Integrated microRNA and mRNA expression profiling reveals a complex network regulating pomegranate (*Punica granatum* L.) seed hardness

**DOI:** 10.1038/s41598-018-27664-y

**Published:** 2018-06-18

**Authors:** Xiang Luo, Da Cao, Jianfeng Zhang, Li Chen, Xiaocong Xia, Haoxian Li, Diguang Zhao, Fuhong Zhang, Hui Xue, Lina Chen, Yongzhou Li, Shangyin Cao

**Affiliations:** 1 Zhengzhou Fruit Research Institute, Chinese Academy of Agricultural Sciences, Zhengzhou, 450009 P.R. China; 2 Zhengzhou Tobacco Research Institute of CNTC, Zhengzhou, 450001 P.R. China; 3 National Key Laboratory of Crop Genetic Improvement, National Center of Rapeseed Improvement in Wuhan, Huazhong Agricultural University, Wuhan, 430070 P.R. China; 4 College of Horticultural Science, Henan Agricultural University, Zhengzhou, 450002 P.R. China

## Abstract

The breeding of new soft-seeded pomegranate cultivars provides new products for the market and increases farmers’ incomes, yet the genetic architecture mediating seed hardness is largely unknown. Here, the seed hardness and hundred-seed weights of 26 cultivars were determined in 2 successive years. We conducted miRNA and mRNA sequencing to analyse the seeds of two varieties of *Punica granatum*: soft-seeded Tunisia and hard-seeded Sanbai, at 60 and 120 d after flowering. Seed hardness was strongly positively correlated with hundred-seed weight. We detected 25 and 12 differentially expressed miRNA–mRNA pairs with negative regulatory relationships between the two genotypes at 60 and 120 d after flowering, respectively. These miRNA–mRNA pairs mainly regulated seed hardness by altering cell wall structure. Transcription factors including NAC1, WRKY and MYC, which are involved in seed hardness, were targeted by differentially expressed mdm-miR164e and mdm-miR172b. Thus, seed hardness is the result of a complex biological process regulated by a miRNA–mRNA network in pomegranate. These results will help us understand the complexity of seed hardness and help to elucidate the miRNA-mediated molecular mechanisms that contribute to seed hardness in pomegranate.

## Introduction

Pomegranate is an edible fruit that is native to central Asia^1^. It has gained attention because of its antioxidant properties that have health benefits for humans and protect against several diseases such as hypertension cardiovascular and cancer^2^. Pomegranate seeds contain phytosterols and have a special fatty acid profile that includes punicic acid, which contributes to their health benefits^3^. Pomegranates with seeds that are easy to swallow command higher market prices than traditional varieties. In China, the best-known commercial soft-seeded pomegranate variety is Tunisia. This variety has been cultivated in China for more than 30 years^4^, resulting in cultivar depression. Thus, breeding new soft-seeded cultivars is imperative to meet market demands. Fully characterising the genetic mechanism of seed hardness may be useful to breed new commercially viable pomegranate varieties.

A few recent studies have focused on the seed hardness of pomegranate. Our previous study showed that the seed hardness increased from 60 to 120 d after flowering (DAF) in hard-seeded varieties, but did not change during this period in soft-seeded varieties. Additionally, the latter had lower lignin contents than the former^4^. These results were consistent with those of Zarein *et al*.^5^, in which soft-seeded pomegranate was found to have a higher cellulose content than that of hard-seeded pomegranate at 60 and 120 DAF. Correspondingly, lignin and cellulose biosynthetic genes such as *CCR*, *CAD*, *CelSy*, *SuSy*, *CCoA-OMT*, *MYB*, *WRKY* and *MYC*, showed differences in their seed expression levels between soft- and hard-seeded pomegranate genotypes^4,5^. In another study, four quantitative trait loci (QTLs) associated with seed hardness could explain 15% to 30% of the phenotypic variation^6^. These results indicated that seed hardness involves complicated physiological processes and is controlled by multiple genetic factors. However, none of the reported genes or QTLs could completely explain the genetic basis of seed hardness in pomegranate.

Genes that determine important traits are usually negatively regulated by microRNAs (miRNAs), small noncoding RNAs of 18–25 nucleotides (nt), through either posttranscriptional degradation or translational repression^7^. The advent of next-generation sequencing has revealed many miRNAs in plants and highlighted their differential expression levels in different plant species with phenotypic variations. miRNAs are known to participate in numerous biological processes related to human diseases, such as cancer and metabolic diseases^8^. They also participate in plant growth, development, and stress responses^7,9^. A recent study identified miRNAs from 10-d-old seedlings, leaves, flowers and arils of pomegranate at different developmental stages^10^. Both conserved and pomegranate-specific miRNAs were identified, and of the former, the most abundant miRNA family was miR157. Bioinformatics analysis revealed that the miRNAs were mainly involved in regulating linolenic and ascorbate acid metabolism, sugar metabolism, RNA transport, and plant hormone signalling. However, the roles of miRNAs or miRNA targets during seed development, especially related to the development of seed hardness, are yet to be elucidated in pomegranate.

In the study, we conducted deep-sequencing and bioinformatics analysis of seeds at 60 and 120 DAF to identify pomegranate-specific miRNAs, and to determine their expression patterns, in two varieties of *P. granatum*: soft-seeded Tunisia and hard-seeded Sanbai. The identification of these differentially expressed miRNAs–mRNAs provides new insights into the genetic mechanism of seed hardness in pomegranate.

## Results

### Phenotypic variations and correlation analysis

**Figure 1:**
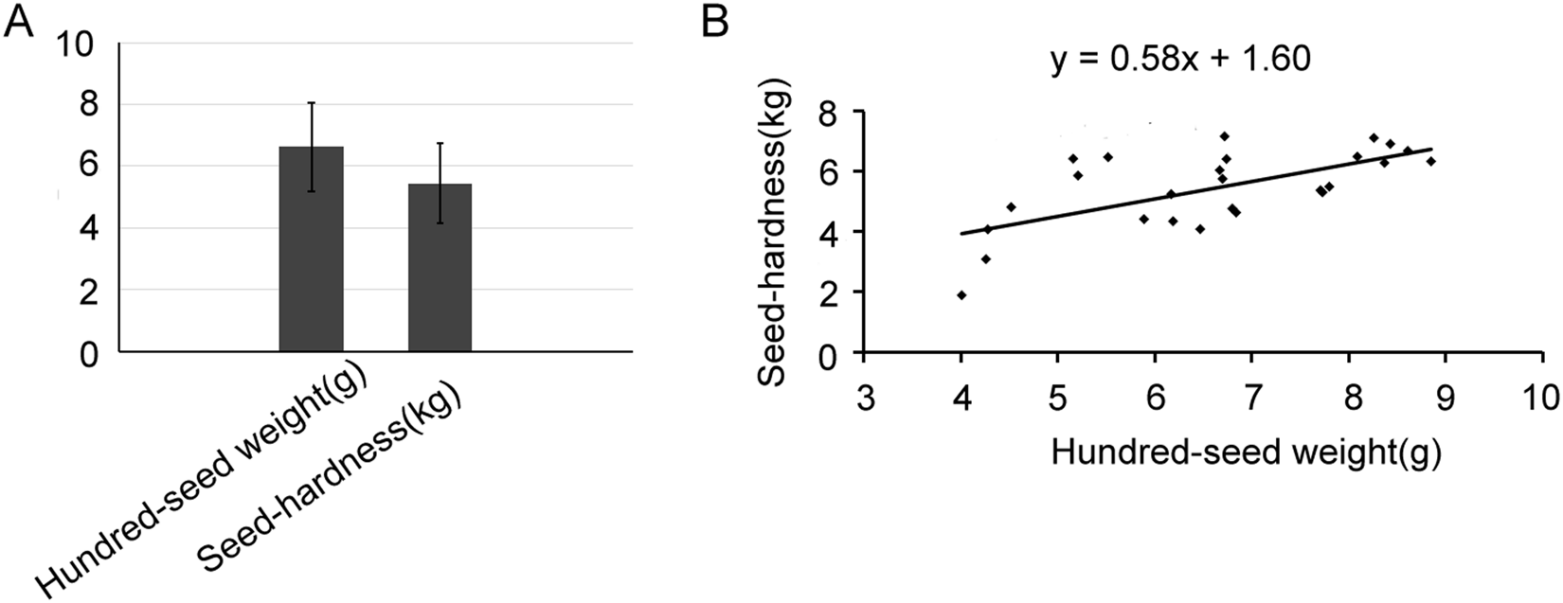
Phenotypic variation (**A**) and correlation (**B**) analyses of seed hardness and hundred-seed weight in pomegranate.

Seed weight is a key factor that controls seed size, and ‘Tunisia’ seeds are smaller and lighter in weight than those of ‘Sanbai’^4^. To explore the relationship between seed weight and seed hardness, phenotypic variations in hundred-seed weight and seed hardness were analysed for 26 pomegranate cultivars (Supplementary Table S1). The average hundred-seed weight and seed hardness of the cultivars were 6.62 and 5.44, respectively (Fig. 1A). Seed hardness was positively correlated with hundred-seed weight (correlation coefficient, 0.58; *P* < 0.01) (Fig. 1B). A linear regression analysis of the correlated traits indicated that hundred-seed weight could significantly explain 40.24% of the seed hardness (*P* < 0.01).

### Construction and deep sequencing of small RNAs

The total RNAs were isolated from the seeds of ‘Tunisia’ and ‘Sanbai’ at 60 and 120 DAF. These RNAs were used to construct four small RNA libraries; SS1, SS2, TS1, and TS2 (Supplementary Table S2), and small RNA sequencing generated 24,593,968, 24,362,778, 24,221,594, and 24,222,409 raw reads from these respective libraries. The 5′ and 3′ adaptors and low-quality reads were removed from the raw reads, yielding 23,025,209, 22,362,305, 22,879,827, and 22,523,261 reads from SS1, SS2, TS1, and TS2, respectively. Approximately 93.99%, 95.51%, 91.35%, and 90.68% of the clean reads from SS1, SS2, TS1, and TS2, respectively, were successfully mapped to the reference genome. Among them, reads with sequence lengths of 18–30 nt were filtered to enrich the sample with reads corresponding to the size of typical small RNAs. The distribution of small RNA lengths among the different size categories is shown in Supplementary Fig. S1. In SS1 and TS1, 23- and 24-nt were the most abundant sizes, while in SS2 and TS2, the most abundant small RNAs were 21 and 24 nt in length. Thus, the 24-nt small RNAs dominated the small RNA transcriptome of all of the libraries.

To identify miRNAs from the four small RNA libraries, we used sequence tags of 18–30 nt in length as queries in BLAST searches against the miRBase^11^ and Rfam^12^ databases. We identified 207 known miRNAs belonging to 40 miRNAs families (Supplementary Table S3). Among the conserved miRNAs detected, the MIR156 family was the largest, with 31 members (14.98% of the total). The MIR171_1 and MIR172 families contained 15 members each. MIR399 and MIR167_1 both included 10 members, and MIR166 and MIR395 had nine members each. The MIR1511, MIR477, MIR7125, and MIR827_4 families each had a single member. Five known miRNAs (mdm-miR391, mdm-miR477a, mdm-miR7126, mdm-miR7128, and mdm-miR858), could not be assigned to existing miRNA families, indicating that they may be species-specific or present in only some plant species. We used the miRNA prediction software Mireap^13^ to obtain putative novel miRNAs with their predicted hairpin precursors. By exploring the secondary structure, dicer cleavage sites, and the minimum free energy of the un-annotated small RNA tags that could be mapped to the reference genome, 761 potential novel miRNA candidates were identified from all four libraries (Supplementary Table S4). The putative precursor sequences of these predicted miRNAs were further analysed using RNAfold software to confirm their stem-loop structures. All the precursor sequences folded into hairpin-like structures that were similar to those of other known miRNAs (Supplementary Fig. S2). In total, 830 (137 known and 693 novel), 745 (135 known and 610 novel), 850 (145 known and 705 novel) and 795 (136 known and 659 novel) miRNAs were detected in the SS1, SS2, TS1, and TS2 libraries, respectively (Supplementary Table S2).

### Differential expression analysis of miRNAs

miRNAs with least 10 raw read counts from the four equivalent libraries were selected for further analysis. We identified differentially expressed miRNAs between SS1_TS1 and SS2_TS2. Compared with the SS group, the TS group had 94 (31 known and 63 novel) and 120 (26 known and 94 novel) significantly differentially expressed miRNAs at 60 and 120 DAF, respectively, based on the criteria |log _2_ (TS/SS)| ≥ 1 and *P* ≤ 0.01 (Supplementary Table S5).

**Figure 2:**
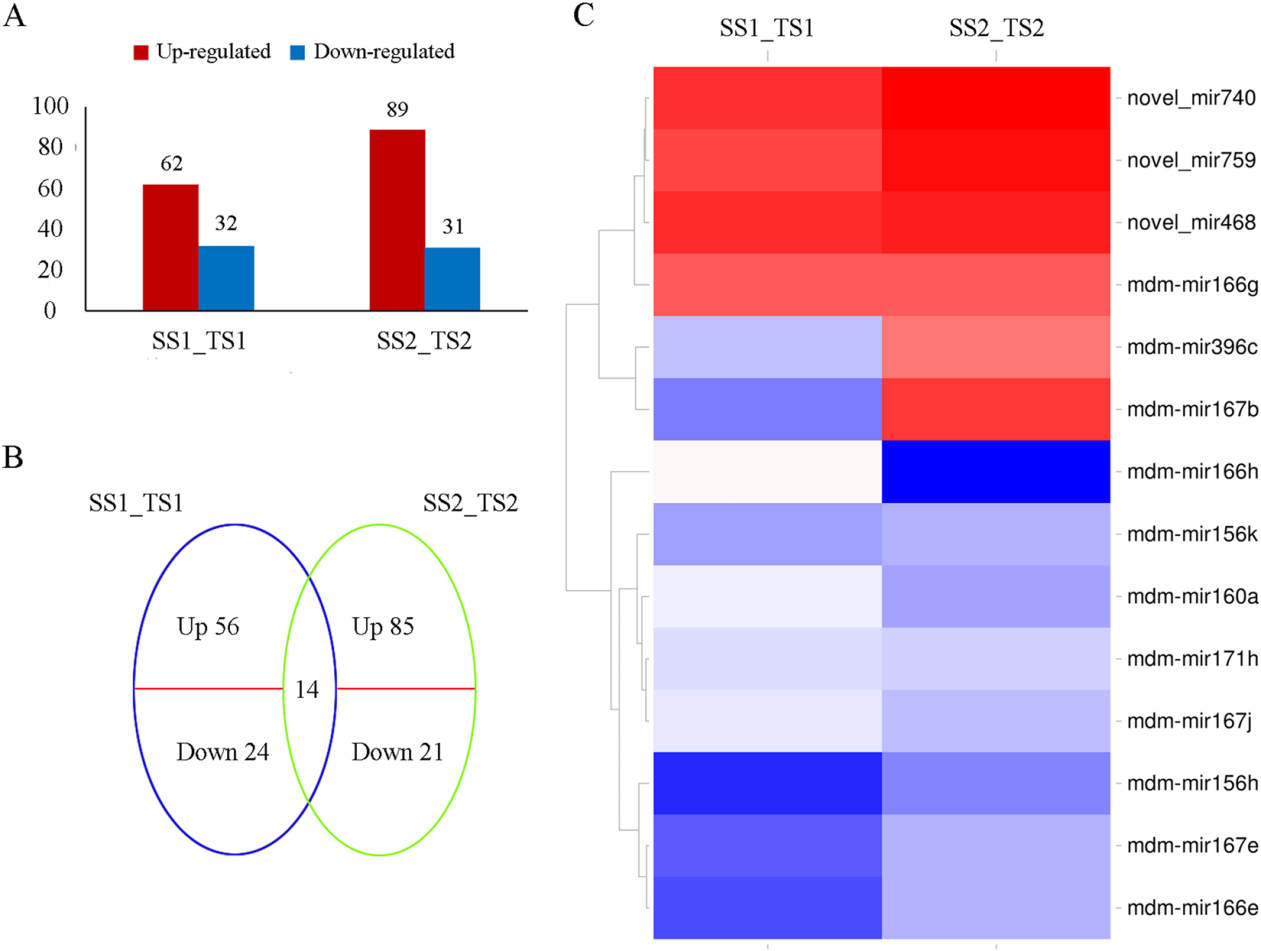
Differentially expressed miRNAs in pomegranate seed. (**A**) Numbers of miRNAs up- or down-regulated in SS1_TS1 and SS2_TS2. (**B**) Venn diagram showing unique and shared regulatory miRNAs in SS1_TS1 and SS2_TS2. (**C**) Hierarchical cluster analysis of 14 regulated miRNAs in SS1_TS1 and SS2_TS2. Fold-change ratios of genes are indicated by different colours. SS1_TS1: comparison between seeds of ‘Sanbai’ and ‘Tunisia’ at 60 d after flowering (DAF). SS2_TS2: comparison between seeds of ‘Sanbai’ and ‘Tunisia’ at 120 DAF.

In total, 62 of the 92 differentially expressed miRNAs in SS1_TS1 and 89 of the 120 differentially expressed miRNAs in SS2_TS2 were up-regulated, while all of the others were down-regulated (Fig. 2A). A Venn diagram (Fig. 2B) showed that the relative expression levels of 14 miRNAs changed in both SS1_TS1 and SS2_TS2. In SS1_TS1, eight out of 14 miRNAs were down-regulated, while in SS2_TS2 10 out of 14 miRNAs were down-regulated (Fig. 2C). Of the 14 common differentially expressed miRNAs, three novel miRNAs were up-regulated in both SS1_TS1 and SS2_TS2. Eighty (56 up-regulated and 24 down-regulated) miRNAs were specifically expressed in SS1_TS1, while 106 (85 up-regulated and 21 down-regulated) miRNAs were specifically expressed in SS2_TS2. The large number of differentially expressed miRNAs may contribute to differences in seed development between the two genotypes. The stage-specific differentially expressed miRNAs exhibited time–space specificity in pomegranate.

### Correlation analysis of miRNAs and their target mRNAs

We used psRobot^14^ and TargetFinder^15^ with position-dependent scoring systems to predict the miRNA targets. These analyses identified 2,646 putative targets; 2,016 miRNA–mRNA pairs identified by psRobot and 1,795 pairs identified by TargetFinder, with 1,165 being common to both (Supplementary Fig. S3). Differences in the scoring matrices of the software explained the discrepancy in the number of targets predicted between the two programs. In total, 408 and 335 miRNA–mRNA pairs were identified in SS1_TS1 and SS2_TS2, respectively (Supplementary Table S6).

**Figure 3:**
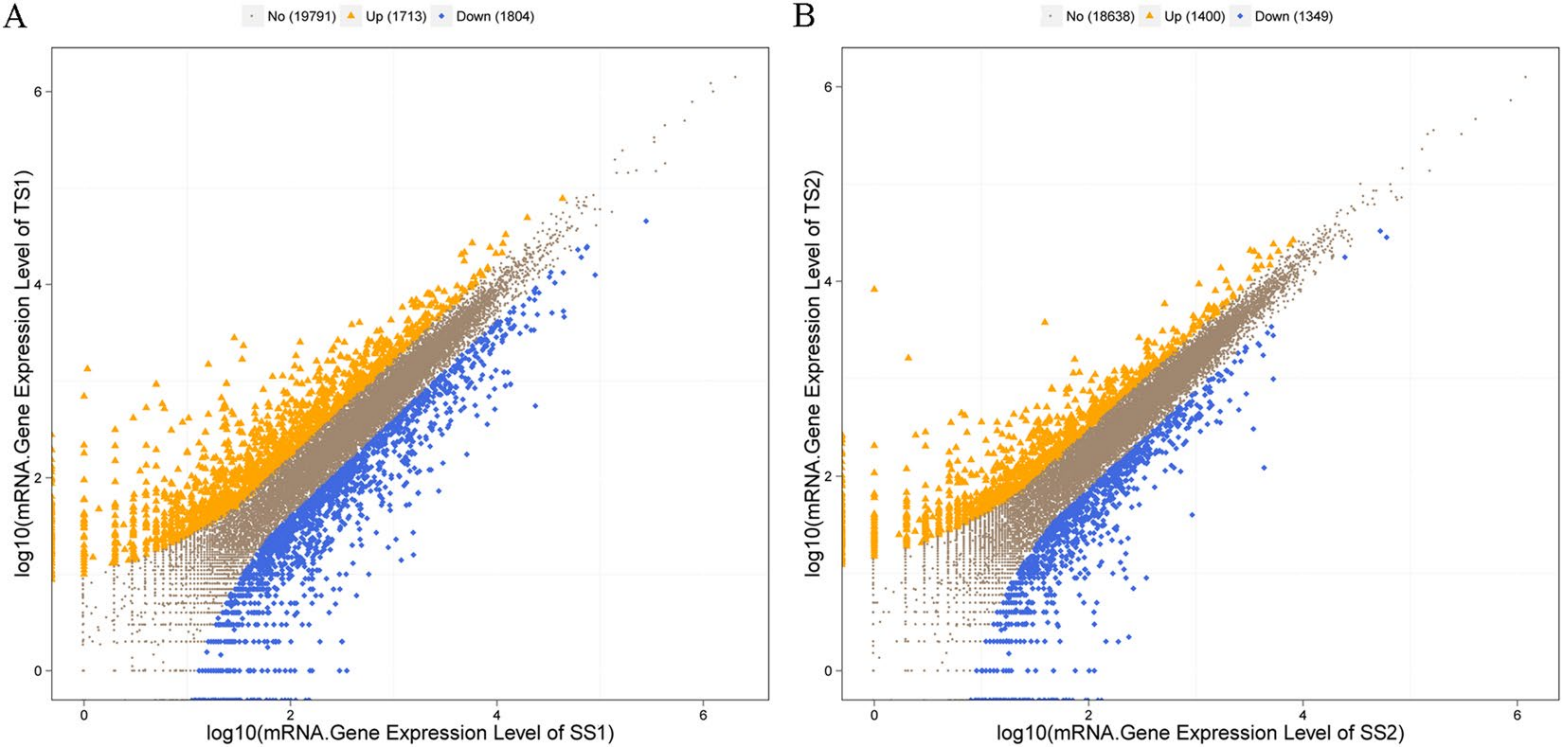
Identification of differentially expressed genes in (**A**) SS1_TS1 and (**B**) SS2_TS2.

Previously, we performed *de novo* assembly of the seed transcriptome^4^, and sequenced and assembled the pomegranate genome (unpublished data). Thus, the sequence tags were re-annotated based on our reference genome. Differentially expressed genes between the two varieties were identified by Cuffdiff based on the criteria *P* ≤ 0.01 and |log_2_ (TS/SS)| ≥ 1. In this way, we identified 1,713 up-regulated and 1,804 down-regulated genes in SS1_TS1, and 1,400 up-regulated and 1,349 down-regulated genes in SS2_TS2 (Fig. 3). Combined with the differentially expressed miRNA targets, 53 and 38 differentially expressed miRNA–mRNA pairs were independently acquired from SS1_TS1 and SS2_TS2, respectively (Supplementary Table S7).

**Figure 4:**
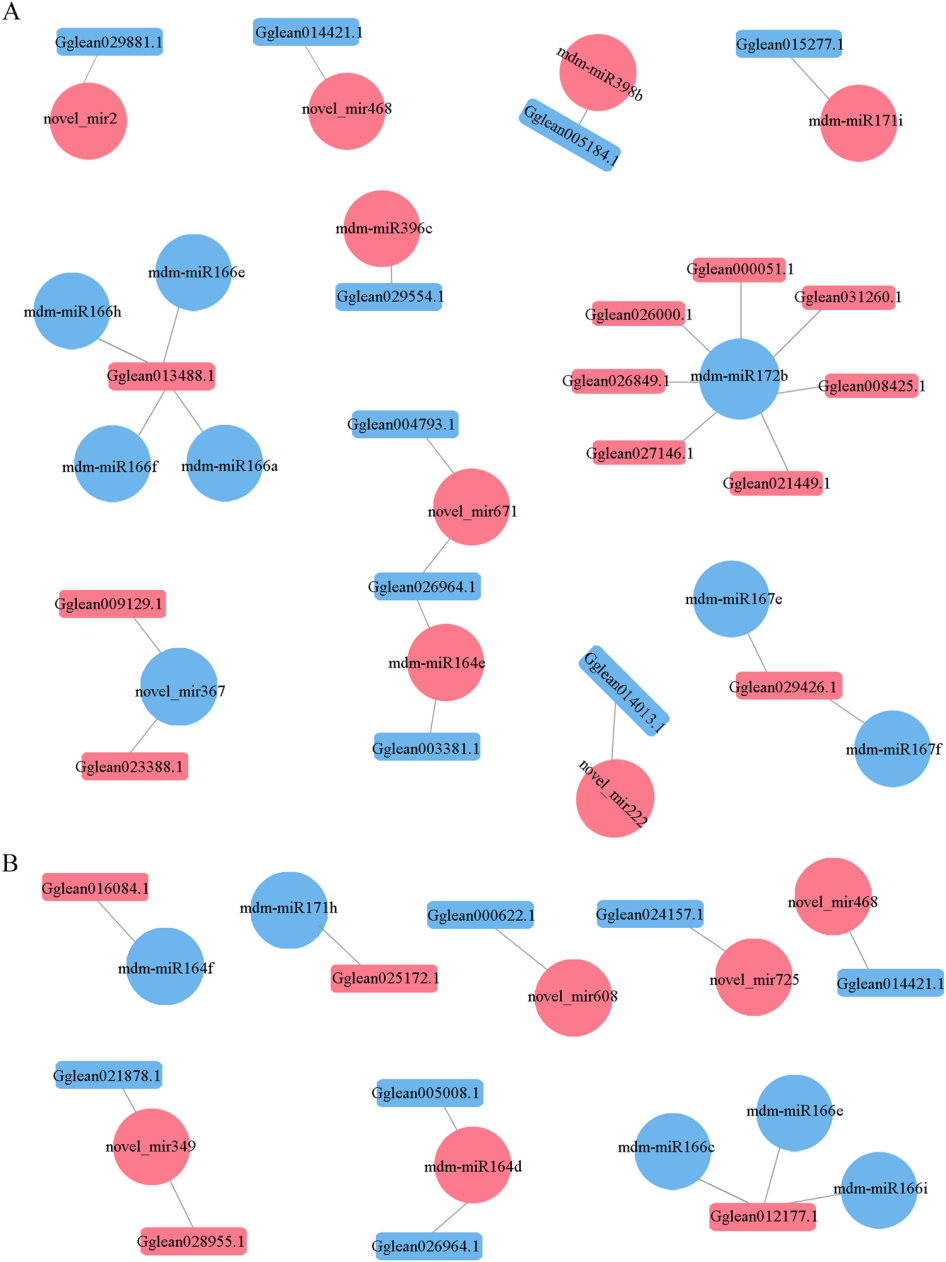
Combined analysis of negative regulatory miRNA and mRNA expression networks in (**A**) SS1_TS1 and (**B**) SS2_TS2. Rectangles represent mRNAs; circles represent miRNAs; blue and red represent up- and down-regulated, respectively.

Usually, miRNAs regulate their targets by inducing mRNA degradation^16,17^. Microarray analyses have shown that miRNA expression decreases the abundance of transcripts with potential miRNA target sites^18–20^. Thus, to explore the roles of miRNA and mRNA interactions in seed hardness, we studied the negative regulatory relationships between miRNAs and their targets (Table 1). In total, 25 and 12 miRNA–mRNA pairs were identified in SS1_TS1 and SS2_TS2, respectively. The relationships between differentially expressed miRNAs and mRNAs were grouped into three major classes based on their regulatory modes (Fig. 4): (1) one miRNA versus one mRNA; (2) one miRNA or mRNA versus more than one mRNA or miRNA; and (3) one miRNA mediating the expression of another miRNA. An example of type 2 was mdm-miR172b, which repressed *Gglean031260.1*, *Gglean008425.1*, *Gglean027146.1*, *Gglean026849.1*, *Gglean026000.1*, and *Gglean000051.1*. *Gglean013488.1* was simultaneously negatively regulated by mdm-miR166h, mdm-miR166e, mdm-miR166f, and mdm-miR166a in the MIR166 family. An example of type 3 was novel_mir671, which negatively regulated *Gglean004793.1*. Intriguingly, the expression of novel_mir671 and its precursors may be directly regulated by *Gglean026964.1*, the target gene of mdm-miR164e. This indicated that novel_mir671 and mdm-miR164e together target *Gglean026964.1* to contribute to the seed hardness of pomegranate. Only one of the miRNA–mRNA interaction pairs (novel_mir468–*Gglean014421.1*) was acquired from both SS1_TS1 and SS2_TS2. Together, these results highlighted the complicated interactions between miRNAs and mRNAs during seed development in pomegranate.

**Table 1:** Predicted mRNA targets of differentially expressed miRNAs in SS1_TS1 and SS2_TS2.

miRNA id	*P* value	log2(TS/SS)	Regulation	Target gene id	*P* value	log2(TS/SS)	Regulation
**SS1_TS1**							
mdm-miR171i	0.000	4.291	Up	Gglean015277.1	0.001	−2.792	Down
novel_mir222	0.001	1.691	Up	Gglean014013.1	0.000	−1.164	Down
mdm-miR166h	0.000	−8.224	Down	Gglean013488.1	0.000	1.564	Up
mdm-miR166e	0.000	−3.634	Down	Gglean013488.1	0.000	1.564	Up
mdm-miR166f	0.000	−2.458	Down	Gglean013488.1	0.000	1.564	Up
mdm-miR166a	0.000	−6.320	Down	Gglean013488.1	0.000	1.564	Up
mdm-miR167e	0.001	−3.653	Down	Gglean029426.1	0.000	7.351	Up
mdm-miR167f	0.001	−2.773	Down	Gglean029426.1	0.000	7.351	Up
mdm-miR172b	0.002	−4.193	Down	Gglean031260.1	0.000	1.263	Up
mdm-miR172b	0.002	−4.193	Down	Gglean008425.1	0.000	5.103	Up
mdm-miR172b	0.002	−4.193	Down	Gglean021449.1	0.001	2.996	Up
mdm-miR172b	0.002	−4.193	Down	Gglean027146.1	0.000	2.122	Up
mdm-miR172b	0.002	−4.193	Down	Gglean026849.1	0.000	1.604	Up
mdm-miR172b	0.002	−4.193	Down	Gglean026000.1	0.000	1.544	Up
mdm-miR172b	0.002	−4.193	Down	Gglean000051.1	0.000	9.279	Up
mdm-miR398b	0.007	3.374	Up	Gglean005184.1	0.000	−1.389	Down
novel_mir671	0.000	3.853	Up	Gglean004793.1	0.000	−1.151	Down
novel_mir671	0.000	3.853	Up	Gglean026964.1	0.000	−2.145	Down
mdm-miR164e	0.007	3.247	Up	Gglean026964.1	0.000	−2.145	Down
novel_mir2	0.001	2.774	Up	Gglean029881.1	0.000	−2.219	Down
novel_mir367	0.000	−2.738	Down	Gglean009129.1	0.000	1.045	Up
novel_mir367	0.000	−2.738	Down	Gglean023388.1	0.000	1.194	Up
novel_mir468	0.002	4.128	Up	Gglean014421.1	0.000	−2.026	Down
mdm-miR164e	0.007	3.247	Up	Gglean003381.1	0.000	−4.952	Down
mdm-miR396c	0.005	1.832	Up	Gglean029554.1	0.001	−1.996	Down
**SS2_TS2**							
novel_mir608	0.000	5.483	Up	Gglean000622.1	0.000	−7.608	Down
novel_mir349	0.001	3.834	Up	Gglean028955.1	0.000	−2.365	Down
novel_mir468	0.002	3.810	Up	Gglean014421.1	0.000	−1.571	Down
novel_mir725	0.000	4.828	Up	Gglean024157.1	0.000	−1.443	Down
mdm-miR164d	0.000	3.948	Up	Gglean005008.1	0.000	−1.411	Down
novel_mir349	0.001	3.834	Up	Gglean021878.1	0.000	−1.242	Down
mdm-miR164d	0.000	3.948	Up	Gglean026964.1	0.000	−1.100	Down
mdm-miR171h	0.000	−2.538	Down	Gglean025172.1	0.000	1.128	Up
mdm-miR166e	0.000	−6.325	Down	Gglean012177.1	0.000	1.775	Up
mdm-miR166c	0.002	−3.015	Down	Gglean012177.1	0.000	1.775	Up
mdm-miR166i	0.000	−5.615	Down	Gglean012177.1	0.000	1.775	Up
mdm-miR164f	0.001	−3.826	Down	Gglean016084.1	0.000	2.355	Up

### Validation of miRNA and mRNA expression

**Figure 5:**
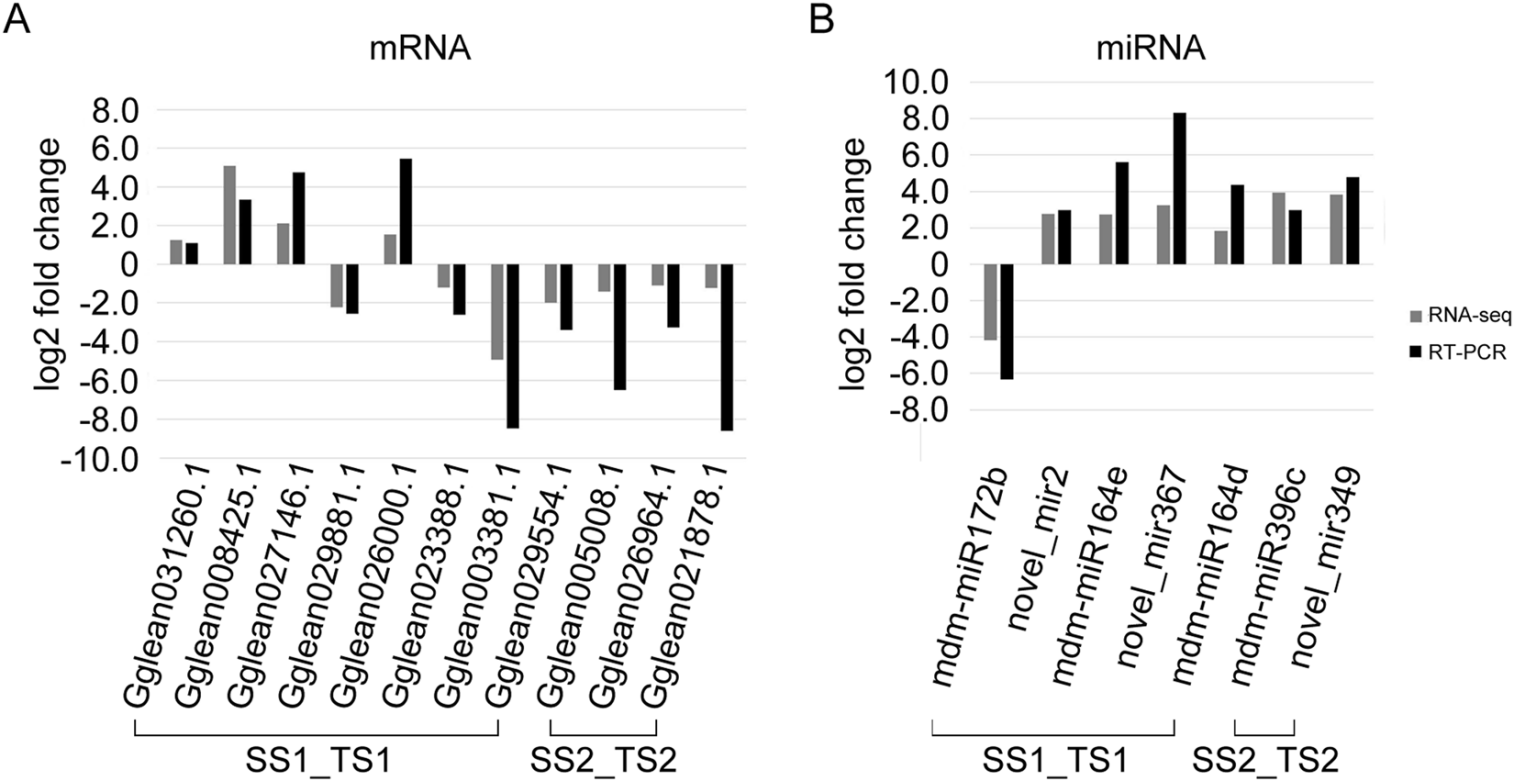
qRT-PCR analysis of selected (**A**) mRNAs and (**B**) miRNAs.

To further confirm the miRNA and mRNA sequencing results, a qRT-PCR analysis was used to validate the expression patterns of the differentially expressed miRNAs and their targets. Seven miRNAs (mdm-miR172b, novel_mir2, novel_mir367, mdm-miR164e, mdm-miR396c, mdm-miR164d, and novel_mir349) and their targets were selected for these analyses. The results of the qRT-PCR analyses were very similar to those obtained from the high-throughput sequencing data (Fig. 5).

### GO analysis of targets of differentially expressed miRNAs

**Figure 6:**
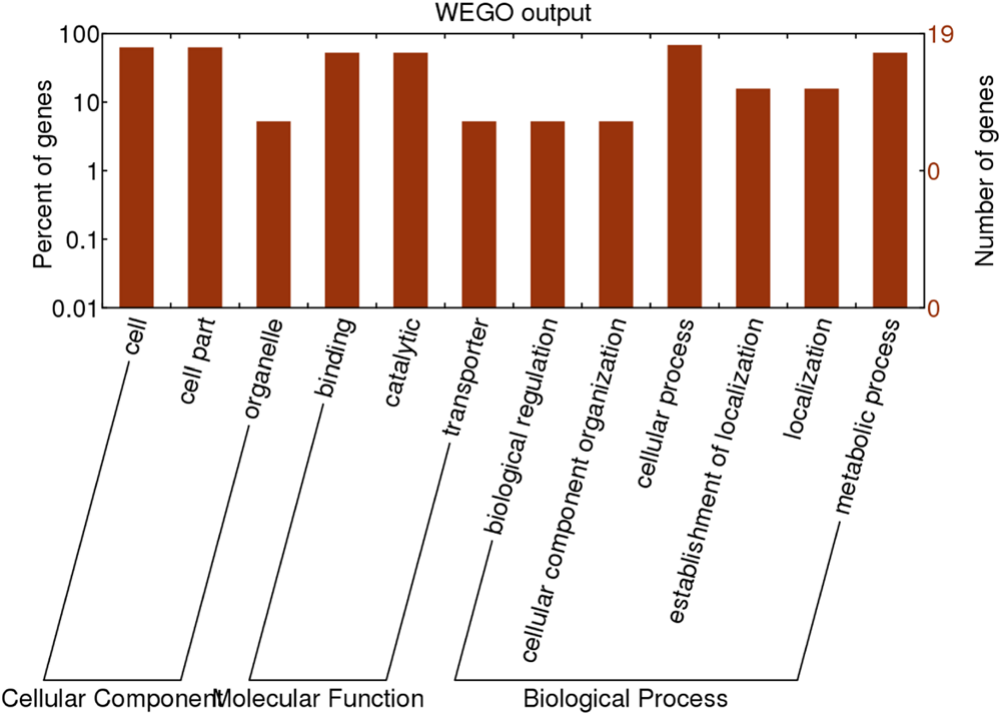
GO analysis of differentially expressed miRNA targets.

To determine the functions of the targets of the differentially expressed miRNAs, we conducted GO analyses of the predicted targets using a false discovery rate correction value at *P* ≤ 0.05. These analyses showed that ‘cellular component’, ‘molecular function’ and ‘biological process’ were the three main categories enriched with targets of the differentially expressed miRNAs between TS and SS pomegranate (Fig. 6). In the ‘cellular components’ category, genes related to cell, cell part, and organelle were highly enriched as targets of the differentially expressed miRNAs. In the ‘molecular functions’ category, genes related to binding, catalytic activity, and transporter activity were highly enriched as targets of the differentially expressed miRNAs. In the ‘biological processes’ category, genes targeted by differentially expressed miRNAs were involved in biological regulation, cellular component organisation, cellular process, establishment of localisation, and localisation and metabolic process. Cellular process- and metabolic process-related genes were significantly overrepresented in the ‘biological processes’ category, indicating that these two processes were greatly enhanced.

### KEGG pathway analysis of differentially expressed miRNA targets

**Figure 7:**
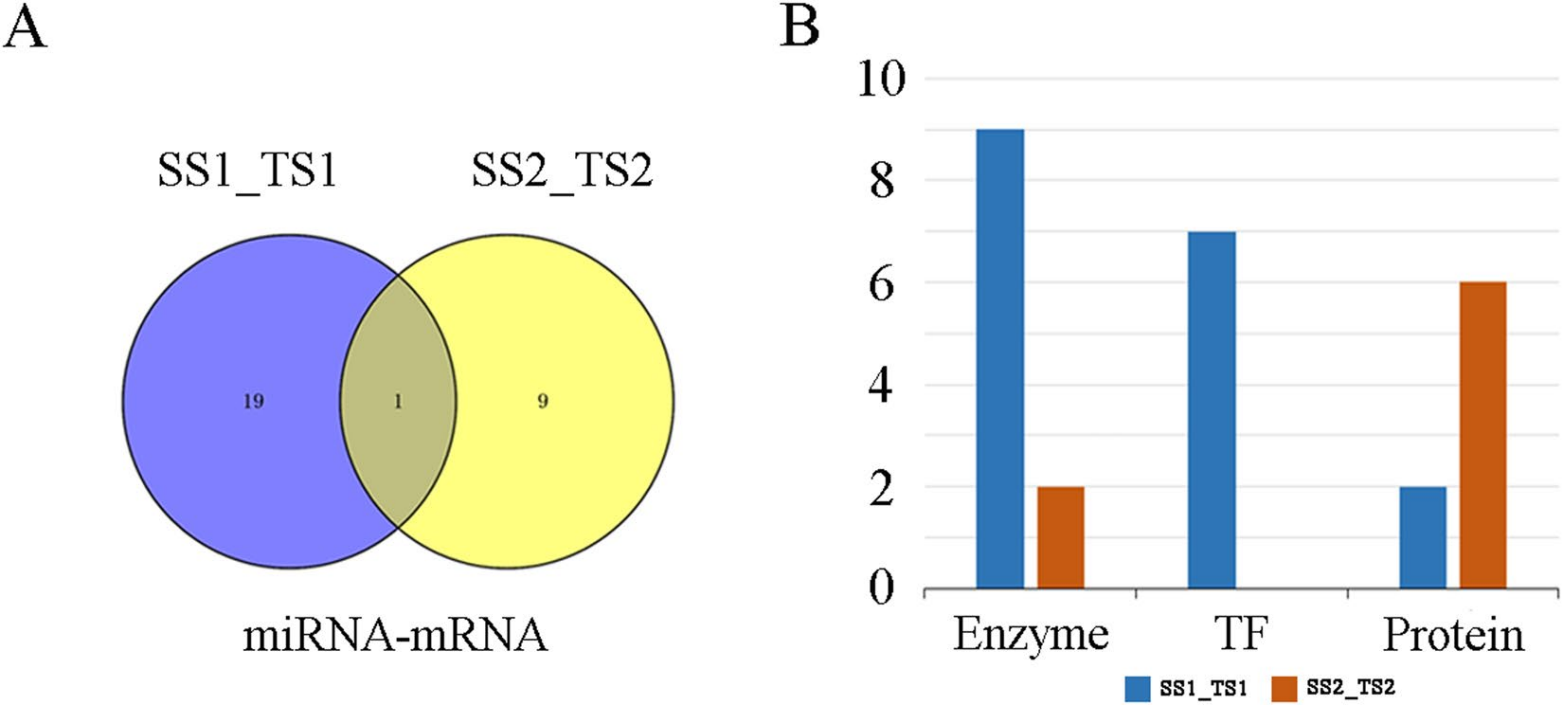
KEGG pathway analysis of differentially expressed miRNA–mRNAs. (**A**) Venn diagram showing unique and shared differentially expressed miRNA–mRNAs acting in pathways in SS1_TS1 and SS2_TS2. (**B**) Distributions of pathways related to enzymes, transcription factors, and proteins in SS1_TS1 and SS2_TS2.

To further explore the functions of differentially expressed miRNAs targets, we conducted KEGG pathway and comparative analyses (Table 2). A total of 14 and eight differentially expressed miRNAs targets were assigned to 18 and eight pathways in SS1_TS1 and SS2_TS2, respectively. Twenty and 10 differentially expressed miRNA–mRNA pairs were detected as acting in these pathways in SS1_TS1 and SS2_TS2, respectively (Fig. 7A and Table 2). Of those, only one pair (novel_mir468-Gglean014421.1) was co-expressed in SS1_TS1 and SS2_TS2 (Fig. 7A and Table 2). We hypothesise that these miRNA–mRNA pairs play important roles in the development of seed hardness.

**Table 2:** KEGG pathway analysis of differentially expressed miRNA-mRNAs in SS1_TS1 and SS2_TS2.

Pomegranate					Arabidopsis thaliana	
DE-miRNAs	Regulation	DEGs	Entry	Annotation	Gene	Description
**SS1-TS1**						
mdm-miR171i	Up	*Gglean015277.1*	K08193	solute carrier family 17 (sodium-dependent inorganic phosphate cotransporter)	—	
novel_mir222	Up	*Gglean014013.1*	K00517	indol-3-yl-methylglucosinolate hydroxylase [EC:1.14.-.-]	AT2G46660 (EOD3)	Encodes a member of CYP78A cytochrome P450 monooxygenase
mdm-miR166h	Down	*Gglean013488.1*	K00820	glucosamine–fructose-6-phosphate aminotransferase (isomerizing) [EC:2.6.1.16]	—	
mdm-miR166e						
mdm-miR166f						
mdm-miR166a						
mdm-miR167e	Down	*Gglean029426.1*	K05681	ATP-binding cassette subfamily G (WHITE) member 2	—	
mdm-miR167f						
mdm-miR172b	Down	*Gglean031260.1*	K09284	AP2-like transcription factor	AT2G28550	related to AP2.7
		*Gglean008425.1*	K13424	WRKY transcription factor 33	—	
			K18835	WRKY transcription factor 2	—	
		*Gglean027146.1*	K13422	transcription factor MYC2	—	
		*Gglean026849.1*	K16225	WRKY transcription factor 52	—	
			K13425	WRKY transcription factor 22	—	
		*Gglean026000.1*	K13418	somatic embryogenesis receptor kinase 1, [EC:2.7.10.1 2.7.11.1]	AT3G25560	NSP-interacting kinase 2
		*Gglean000051.1*	K01510	apyrase [EC:3.6.1.5]	—	
novel_mir671	Up	*Gglean026964.1*	K08678	UDP-glucuronate decarboxylase [EC:4.1.1.35]	AT3G46440	encodes a protein similar to UDP-glucuronic acid decarboxylase
novel_mir2	Up	*Gglean029881.1*	K01115	phospholipase D1/2 [EC:3.1.4.4]	—	
novel_mir367	Down	*Gglean009129.1*	K12355	coniferyl-aldehyde dehydrogenase [EC:1.2.1.68]	AT3G24503	aldehyde dehydrogenase AtALDH1a
		*Gglean023388.1*	K01728	pectate lyase [EC:4.2.2.2]	AT1G04680	pectin lyase-like superfamily protein
mdm-miR164e	Up	*Gglean003381.1*	K13126	NAC1 transcription factor	AT1G56010 (NAC1)	encodes a NAC
mdm-miR396c	Up	*Gglean029554.1*	K05275	pyridoxine 4-dehydrogenase [EC:1.1.1.65]	—	
**SS2-TS2**						
mdm-miR166e	Down	*Gglean012177.1*	K09338	homeobox-leucine zipper protein	—	
mdm-miR166c	Down					
mdm-miR166i	Down					
novel_mir608	Up	*Gglean000622.1*	K16578	CLIP-associating protein 1/2	AT2G20190	encodes a microtubule-associated protein
mdm-miR171h	Down	*Gglean025172.1*	K14494	DELLA protein	—	—
mdm-miR164f	Down	*Gglean016084.1*	K08176	inorganic phosphate transporter	AT5G12460	fringe-like protein (DUF604)
mdm-miR164d	Up	*Gglean005008.1*	K13416	brassinosteroid insensitive 1-associated receptor kinase 1 [EC:2.7.10.1 2.7.11.1]	AT5G16000	NSP-interacting kinase (NIK1), receptor-like kinase
		*Gglean026964.1*	K08678	UDP-glucuronate decarboxylase [EC:4.1.1.35]	AT3G46440	encodes a protein similar to UDP-glucuronic acid decarboxylase
novel_mir349	Up	*Gglean021878.1*	K05658	ATP-binding cassette	—	
		*Gglean028955.1*	K14794	ribosomal RNA-processing protein 12	—

Eleven miRNA targets (*Gglean014013.1*, *Gglean013488.1*, *Gglean026000.1*, *Gglean000051.1*, *Gglean026964.1*, *Gglean029881.1*, *Gglean009129.1*, *Gglean023388.1*, *Gglean029554.1*, *Gglean005008.1*, *Gglean026964.1*, and *Gglean021878.1*) were annotated as encoding key enzymes that regulate seed hardness in pomegranate (Fig. 7B and Table 2). For example, *Gglean026964.1* encodes UDP-glucuronate decarboxylase [EC: 4.1.1.35], which catalyses the production of UDP-xylose. Its ortholog in *Arabidopsis thaliana* (*AT3G46440*) encodes a similar protein that produces UDP-xylose, a substrate for many cell-wall carbohydrates, including hemicellulose and pectin. UDP-xylose feedback regulates several cell-wall biosynthetic enzymes.

Five miRNA targets (*Gglean031260.1*, *Gglean008425.1*, *Gglean027146.1*, *Gglean026849.1*, and *Gglean003381.1*) were predicted to regulate seven transcription factors (TFs) that affect the formation of seed hardness in pomegranate (Fig. 7B and Table 2). For example, *Gglean008425.1* and *Gglean027146.1* encode proteins in the WRKY family, which includes WRKY2, WRKY33, WRKY22, and WRKY52. *Gglean026849.1* was annotated as MYC2. *Gglean031260.1* and its ortholog in *A. thaliana* (*AT2G2855*) likely have the same function, i.e., to regulate AP2 TFs.

Eight miRNA targets (*Gglean015277.1*, *Gglean029426.1*, *Gglean003381.1*, *Gglean012177.1*, *Gglean000622.1*, *Gglean025172.1*, *Gglean016084.1* and *Gglean028955.1*) were annotated as encoding regulatory proteins (Fig. 7B and Table 2), including a phosphate transporter, ATP-binding domain, polyadenylate-binding, zipper, CLIP-associating, DELLA, and ribosomal RNA-processing protein. These differentially expressed miRNAs may target their corresponding genes to control their expression levels, thereby influencing the development of pomegranate seed hardness.

## Discussion

### Phenotypic analysis

After a double-fertilisation event, seed development begins with embryogenesis (cell division), followed by seed maturation (seed filling via accumulation of storage macromolecules) and then desiccation^21^. The size, weight, and hardness of seeds differ among species and among lines of the same species. All three seed characteristics are inter-related. Here, seed hardness was positively correlated with hundred-seed weight, indicating that decreasing the hundred-seed weight in pomegranate will decrease seed hardness.

### Identification of conserved and novel miRNAs in pomegranate seeds

The combination of deep sequencing with a miRNA microarray analysis has revealed that various miRNAs play vital roles throughout the whole seed cycle, including during dormancy modulation^22^, germination^23^, development^24,25^ and maturation^26^. The results of the present study provide information about the regulatory networks of miRNAs involved in pomegranate seed hardness. Forty known miRNA families were identified, compared with the 30 previously identified known miRNA families^10^. Most are conserved in plants, such as *A. thaliana*^27^ and pomegranate^10^. The most abundant family was found to be miR156, like in pear^28^ and apple^29,30^. Previously, it was reported that miR157 is the most abundant family in pomegranate^10^. This difference from our result might be because different tissue types were analysed in that study. We identified 92 (31 known and 63 novel) and 120 (26 known and 94 novel) miRNAs that were differentially expressed in SS1_TS1 and SS2_TS2, respectively, consistent with the finding that miRNAs are more abundant in mature seeds than in developing seeds^26^. Of the differentially expressed miRNAs, 68.49% and 76.67% were newly identified in SS1_TS1 and SS2_TS2, respectively, indicating their potentially important roles in seed development.

### Comparative analysis between miRNAs and mRNAs

A single miRNA generally targets a broad range of mRNAs with nearly complementary sequences, resulting in the regulation of a wide range of genes^31^. Consequently, miRNAs affect a wide range of physiological and developmental processes, including seed development, in plants. Some pre-miRNA and miRNA targets showing differences in abundance were found to be important in *Jatropha* seed development^32^. Similarly, the putative targets of 21 novel and 87 known miRNAs were predicted to be involved in various metabolic and biological processes in developing cotton seeds^33^. In the dry wheat seeds, the target genes of differentially expressed miRNAs between genetically modified and non-genetically modified lines were found to be associated with abiotic stress^34^.

In the present study, miRNA and mRNA sequencing identified 25 and 12 differentially expressed miRNA–mRNA pairs with negative regulatory relationships from SS1_TS1 and SS2_TS2, respectively. Only one of the miRNA–mRNA interaction pairs (novel_mir468–Gglean014421.1) was co-expressed in both of SS1_TS1 and SS2_TS2. Thus, the development of seed hardness mainly relies on the stage-specific expression of miRNA–mRNA pairs in pomegranate. A KEGG pathway analysis indicated that the functions of the targets of the differentially expressed miRNAs were mainly related to regulation of TFs and metabolic enzymes in SS1_TS1, and to structure-related proteins in SS2_TS2 (Table 2). These results were consistent with seed physiology and development. That is, intense metabolic activity depends on enzymatic activity and TFs that act during early stages, when metabolism is mainly involved in carbohydrate partitioning to supply photoassimilates and accumulate storage compounds^21^. However, seed maturation requires the deposition of storage macromolecules, including carbohydrates, lipids, and storage proteins^35^. Thus, the mdm-miRNAs may interact with their corresponding targets to control the production of a diverse range of proteins, metabolic enzymes, and TFs, thus modulating seed hardness in pomegranate.

### miRNAs regulate enzymes involved in seed hardness

Enzymes are critical in regulating seed development because they catalyse metabolic processes such as cell-wall formation^36^, and carbohydrate^37^, hormone^38^ and nitrogen metabolism^39^. Here, a predicted novel_mir367 was found to regulate *Gglean009129.1* and *Gglean023388.1*, encoding coniferyl-aldehyde dehydrogenase [EC: 1.2.1.68] and pectate lyase [EC: 4.2.2.2], respectively, which soften seeds. The two genes showed significantly higher expression levels in soft-seeded cultivars than in hard-seeded cultivars. Coniferyl-aldehyde dehydrogenase participates in the formation of ferulate and sinapate, which impede guaiacyl lignin and syringyl lignin biosynthesis (Supplementary Fig. S4). These pathways are similar to those reported in *Arabidopsis* and rapeseed^40,41^. This result may partly explain why soft-seeded pomegranates produces less lignin than hard-seeded ones^4,5^. Pectate lyase is a depolymerising enzyme that degrades plant cell walls^42^. Decreasing the abundance of pectate lyase transcripts can severely inhibit cell wall loosening during early fibre development^43^. In strawberry and tomato, the suppression of the pectate lyase mRNA during ripening resulted in significantly firmer fruit^44^^,^^45^. Thus, we hypothesised that the novel_mir367 controls the expression of genes related to lignin content and loosens the cell wall, resulting in softer pomegranate seeds.

In soft-seeded pomegranate, mdm-miR164e, novel_mir671, and mdm-miR164d down-regulated *Gglean026964.1*, encoding UDP-glucuronate decarboxylase [EC: 4.1.1.35], thereby reducing the seed hardness. UDP-glucuronate decarboxylase is a key enzyme involved in UDP-xylose biosynthesis, which is required for xylan formation during cell-wall biosynthesis^46^. The overexpression of antisense UDP-glucuronate decarboxylase in tobacco enhanced cellulose biosynthesis in secondary cell walls^47^. Thus, these miRNAs may alter cell-wall structure by increasing the cellulose content, which reduces seed hardness. This partly explains the higher proportion of cellulose in soft-seeded pomegranate than in hard-seeded pomegranate^5^.

The novel_mir222 and mdm-miR164d may affect seed weight, which influences seed hardness. The former regulates indol-3-yl-methylglucosinolate hydroxylase [EC: 1.14.-.-] by suppressing *Gglean014013.1*. In *A. thaliana*, its ortholog encodes a member of CYP78A that controls seed size^48^. We hypothesised that the novel_mir222 controls seed size/weight in pomegranate. The seed weight was significantly correlated with seed hardness in pomegranate (Fig. 1). Seed size appears to be regulated primarily by phytohormones, especially brassinosteroids^49^. Regulators can stimulate cell division and cell elongation under the influence of brassinosteroid insensitive 1-associated receptor kinase 1 [EC: 2.7.10.1 2.7.11.1] (Supplementary Fig. S5). In rice, brassinosteroids were shown to cause grain expansion^50,51^. Here, brassinosteroid insensitive 1-associated receptor kinase 1 was found to be regulated by mdm-miR164d through the negative targeting of *Gglean005008.1*.

### miRNAs regulating TFs involved in seed hardness

We found that mdm-miR164e directly down-regulated NAC1 to block seed hardening, while mdm-miR172b upregulated WRKY, MYC and AP2 TFs to achieve the same result. According to our previous transcriptomic analysis, the differential expression of NAC, WRKY, and MYC TFs is related to hardness in pomegranate^4^, and the trends in their regulation detected in the present study corroborated those results. mdm-miR172b repressed the expression of the AP2-like TF, as previously observed in other studies^29,52^. AP2-like TFs control seed size and seed mass in *Arabdopisis*^53–55^. Thus, mdm-miR172b may regulate the AP2-like TF to control seed size in pomegranate. Similarly, osa-miR172 was shown to regulate grain size in rice^56,57^. It may, therefore, be inferred that mdm-miR172b simultaneously regulates multiple TFs that regulate seed hardness in pomegranate.

### miRNAs regulating proteins involved in seed hardness

The predicted miRNA targets encode a broad range of proteins that participate in seed development^26^. In soft-seeded pomegranate cultivars, mdm-miR164f was found to increase the activity of the inorganic phosphate transporter by up-regulating *Gglean016084.1* (Tables 1 and 2). In a previous study, its increased expression level resulted in an increase in phosphate storage^58^, which should contribute to greater seed weight. However, the seed weights of soft-seeded pomegranate were lower than those of hard-seeded pomegranate. The contradiction can be explained by the fact that phosphate transport in and out of the vacuolar membrane requires biochemical energy^59^. A deficiency in energy may limit the phosphate deposition in seed vacuoles, resulting in soft seeds. These results imply that a moderate lack of phosphate could lead to soft seeds. This would be favourable because it would reduce fertiliser consumption and provide a simple way to produce soft-seeded pomegranates^58^.

In pomegranate, the mdm-miR166e, mdm-miR166c, and mdm-miR166i were found to soften seeds by down-regulating the homeobox-leucine zipper proteins. These proteins are a subset of unique proteins that contain leucine zipper motif-linked homeodomains^60^. Reducing the abundance of homeobox-leucine zipper proteins is likely to inhibit cell elongation^61^ and cell division^62^, which may limit seed hardness.

The novel_mir608 is likely to mediate cell-wall biosynthesis, which affects seed hardness in pomegranate. novel_mir608 was found to negatively regulate *Gglean000622.1*, which encodes a CLIP-associating protein that prevents xyloglucan formation. Consequently, it disrupts the stability of the microtubule cytoskeleton and the cellulose pattern in primary cell walls^63^.

## Conclusions

**Figure 8:**
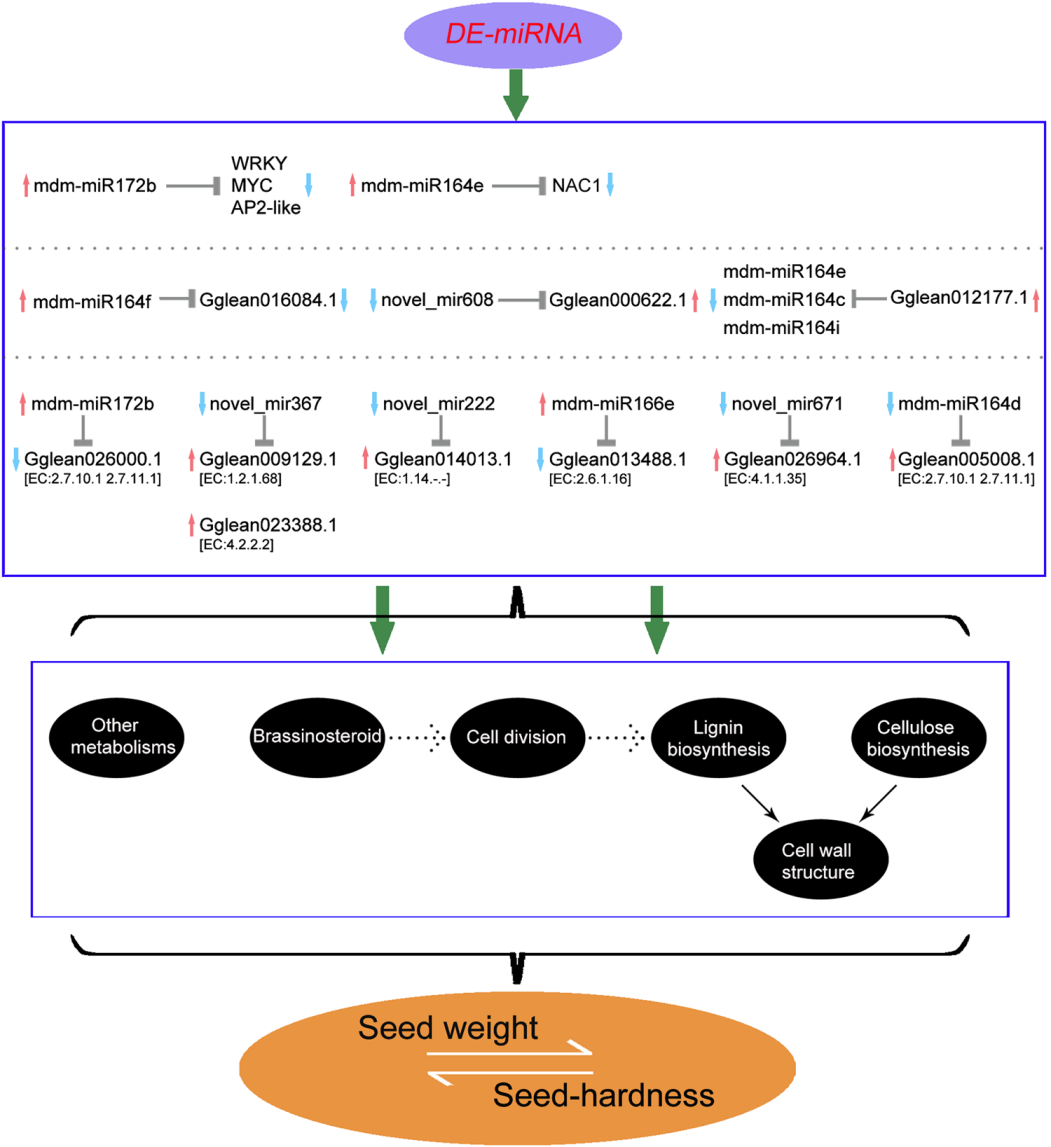
Hypothetical model for regulation of seed hardness in pomegranate through interactions between miRNAs and mRNAs.

We conducted integrated transcriptomics and miRNA analyses to generate a comprehensive resource focused on identifying key regulatory miRNAs associated with the development of seed hardness in pomegranate. We found that these miRNAs suppressed vital targets, which included genes encoding TFs and enzymes involved in the early stages of seed development, as well as proteins involved in storage compound synthesis and transport in the mature seeds. The miRNA-targets included transcripts encoding proteins involved in brassinosteroid biosynthesis, cell elongation and division, lignin biosynthesis, cellulose biosynthesis, cell-wall biosynthesis and degradation, and other metabolic signalling pathways (Fig. 8). The cell wall is mainly composed of lignin and cellulose^64^, and their biosynthesis and degradation affects cell-wall structure. From our results, we concluded that the miRNA targets may regulate seed hardness by altering the cell-wall structure in pomegranate. These findings indicate that seed hardness involves a complex biological process regulated by miRNA–mRNA networks in pomegranate. Other enriched miRNAs were also found during different seed developmental stages and their putative corresponding target genes were investigated. These miRNAs may contribute to seed hardness formation by regulating important metabolic processes, as indicated by the highly represented GO terms (Fig. 6). Our understanding of the genetic relationships among seed weight, seed size, and seed hardness remains limited. Further experiments to confirm the targets of miRNAs and the miRNA–mRNA interaction networks in pomegranate are necessary to test our hypotheses. Collectively, these results will contribute to a more comprehensive understanding of seed hardness in pomegranate and help to elucidate miRNA-mediated molecular mechanisms underlying the formation of seed hardness.

## Methods

### Plant materials and phenotypic analysis

Two pomegranate cultivars, Tunisia (soft-seeded genotype) and Sanbai (hard-seeded genotype), were grown under the same conditions, and were managed in accordance with local standard production practices in Xingyang, China. The fruit developmental periods of the two cultivars were similar. Nine morphologically normal fruits were sampled and pooled in groups of three to obtain three biological replicates from 60 DAF to maturity (120 DAF). All samples were immediately snap frozen in liquid nitrogen and stored at −80 °C. For convenience, the seeds of ‘Tunisia’ and ‘Sanbai’ are abbreviated as TS and SS, respectively. The single seed weights were determined from the average hundred-seed weights. Seed hardness was measured as described by Xue *et al*. (2017). These two traits were studied in 26 cultivars at maturity in 2016 and 2017, in Zhengzhou, China. Both varieties were obtained from the Zhengzhou Fruit Research Institute (Chinese Academy of Agricultural Sciences, Zhengzhou, China).

Phenotypic variation, correlation, and linear regression analyses were performed using SPSS version 19.0 (IBM Corp., Armonk, NY, USA).

### RNA extraction, library construction and deep sequencing

Total RNA was prepared from samples of ‘Tunisia’ and ‘Sanbai’ (control) using TRIzol reagent (Invitrogen, Carlsbad, CA, USA), and the RNA samples were subsequently purified by chloroform extraction. The RNA integrity was checked by electrophoresis on a 0.8% denaturing formaldehyde gel. Only high-quality RNA was used for further analyses. Total RNA (3 mg) was reverse-transcribed to cDNA with RevertAid Reverse Transcriptase (Thermo Fisher Scientific, Waltham, MA, USA) and a random hexamer primer at 42 °C for 60 min. The cDNA was used immediately or stored at −20 °C.

The small RNA libraries were constructed and sequenced using an Illumina Genome Analyzer (Illumina, San Diego, CA, USA) by the BGI Genomics Corporation (Shenzhen, China). Briefly, RNA with integrity >7 was assessed using a Bioanalyzer 2100 (Agilent Technologies, Palo Alto, CA, USA) on an RNA 6000 Nanochip. For small RNA library construction, ~1 μg total RNA was collected into RNA pools according to the Illumina TruSeq Small RNA library preparation protocol. Then, ~16–30 nt gel fragments were selected and ligated to a pair of adapters at the 5′- and 3′-ends using T_4_ RNA ligase. These small RNAs with adapters were transcribed into cDNA using Super-Script II Reverse Transcriptase (Invitrogen). These cDNAs were subjected to PCR amplification, and then the purified PCR products were sequenced.

### Prediction and identification of known and novel miRNAs

To identify known miRNAs in pomegranate, the miRNA categories were mapped to the reference genome (unpublished data) using AASRA^65^. Alignments with less than two mismatches and more than 16 matches without gaps between the query sequences and known miRNAs were considered. The identified miRNAs were classified into families based on their sequence similarities. The unmatched reads were further processed to predict novel miRNAs using Mireap software^13^.

The characteristic structures of miRNA precursors, including hairpins, secondary structures, and dicer cleavage sites, and the minimum free energy were used to predict novel miRNAs with the Mireap pipeline. The criteria included the ability of miRNAs to fold into the correct secondary structure and the presence of mature miRNAs on one arm of the hairpin precursor. Additionally, the free energy of hybridisation had to be lower than or equal to −18 kcal/mol, and the mature miRNA strand and its complementary strand had to contain 2-nt 3′ overhangs.

### mRNA sequencing data analyses and mRNA–miRNA pair predictions

We previously detected differentially expressed genes between soft-seeded and hard-seeded pomegranate by *de novo* transcriptome sequencing^4^. The sequence data were trimmed by removing adaptor sequences, empty reads, reads with more than 5% unknown nucleotides, low-quality sequences (base quality ≤20), and high Ns (ratio >10%) with Trimmomatic^66^. Clean reads were mapped to the reference genome sequence (unpublished data) using TopHat^67^ with default parameters. The reads were then assembled into transcripts and compared with reference gene models using Cufflinks^68^. Gene expression was quantified using the RNA-Seq by Expectation Maximization software^69^. The data were normalised as fragments per kilobase of transcript per million fragments mapped (FPKM) values^70^. The differences in transcript abundance between the two genotypes were calculated based on the ratio of FPKM values. The false discovery rate control method was used to identify the threshold of the P-value using Cuffdiff in the Cufflinks software package. Only transcripts with *P* ≤ 0.001 and |log_2_(TS/SS)| ≥ 1 were used for further analysis. The relative abundance of a gene or miRNA was calculated as follows: normalised expression = (actual miRNA or gene count/total count of clean reads) × 10^6^. After normalisation, differentially expressed miRNAs between the two pomegranate cultivars were identified based on the criteria *P* ≤ 0.01 and |log_2_(TS/SS)| ≥ 1. We used PairFinder software (BGI Genomics Corporation) to predict potential mRNA–miRNA pairs.

### qRT-PCR validation of miRNAs and their targets

We used stem-loop qRT-PCR to confirm the miRNA expression levels^71^. For selected miRNAs, ~1 μg DNA-free total RNA was hybridised with miRNA-specific stem-loop RT primers. The hybridised molecules were reverse transcribed into cDNAs using a Superscript III kit (Thermo Fisher Scientific). We designed forward miRNA-specific primers for the mature miRNA sequences and used a universal reverse primer for the stem-loop sequences. Reactions were repeated three times for each sample set. Each 20-μL reaction mixture contained 1 μL cDNA, 10 μL 2 × FastStart SYBR Green (Roche) and 0.8 μL forward and reverse primers (TaKaRa, Ohtsu, Japan). The PCR amplification conditions were as follows: 95 °C for 10 s and 60 °C for 30 s. The PCRs were conducted using the StepOnePlus Real-Time PCR System (Applied Biosystems, Foster City, CA, USA). Data were analysed using the 2^−ΔΔCt^ method to calculate relative gene expression^72^. Supplementary Table S8 lists all primers used in the qRT-PCR experiments, including those for miRNAs and their targets.

### GO enrichment and pathway enrichment analyses of miRNA targets

The GO enrichment analysis for differentially expressed miRNA targets was conducted using tools at the Blast2GO website (http://www.blast2go.com). Significantly enriched GO terms (P < 0.05) were displayed using the online tool WEGO website (http://wego.genomics.org.cn). The targets of differentially expressed miRNAs were subjected to a KEGG pathway enrichment analysis using tools at the KOBAS2.0 website (http://kobas.cbi.pku.edu.cn/). To analyse the metabolic pathways and functional classifications of the targets of differentially expressed miRNAs, expression data were mapped to metabolic pathways using MapMan software^73,74^.

## Electronic supplementary material

Supplementary Figures and Tables

Dataset 1

Dataset 2
