# Supplementary material for: Integrated microRNA and mRNA expression profiling reveals a complex network regulating pomegranate (Punica granatum L.) seed hardness
Source: Sci Rep. 2018 Jun 18;8:9292. doi: 10.1038/s41598-018-27664-y (PMC6006261; doi:10.1038/s41598-018-27664-y)
Supplement: Supplementary file 1 — Supplementary Figures and Tables [file 41598_2018_27664_MOESM1_ESM.pdf]

Integrated microRNA and mRNA expression profiling reveals a complex network regulating pomegranate (*Punica granatum* L.) seed-hardness

Xiang Luo[1], Da Cao[1], Jianfeng Zhang[2], Li Chen[3], Xiaocong Xia[1], Haoxian Li[1], Diguang Zhao[1], Fuhong Zhang[1], Hui Xue[1], Lina Chen[1], Yongzhou Li[4] and Shangyin Cao[1*]

[1]Zhengzhou Fruit Research Institute, Chinese Academy of Agricultural Sciences, Zhengzhou 450009, P. R. China.

[2]Zhengzhou Tobacco Research Institute of CNTC, Zhengzhou, 450001, P. R. China.

[3]National Key Laboratory of Crop Genetic Improvement, National Center of Rapeseed Improvement in Wuhan, Huazhong Agricultural University, Wuhan 430070, P. R. China.

[4]College of Horticultural Science, Henan Agricultural University, Zhengzhou 450002, P. R. China.

[*]Correspondence and requests for materials should be addressed to S.C

E-mail: s.y.cao@163.com

Zhengzhou Fruit Research Institute, Chinese Academy of Agricultural Sciences, Zhengzhou 450009, P. R. China

Tel: +86-0371-65330990

Fax: +86-0371-65330963

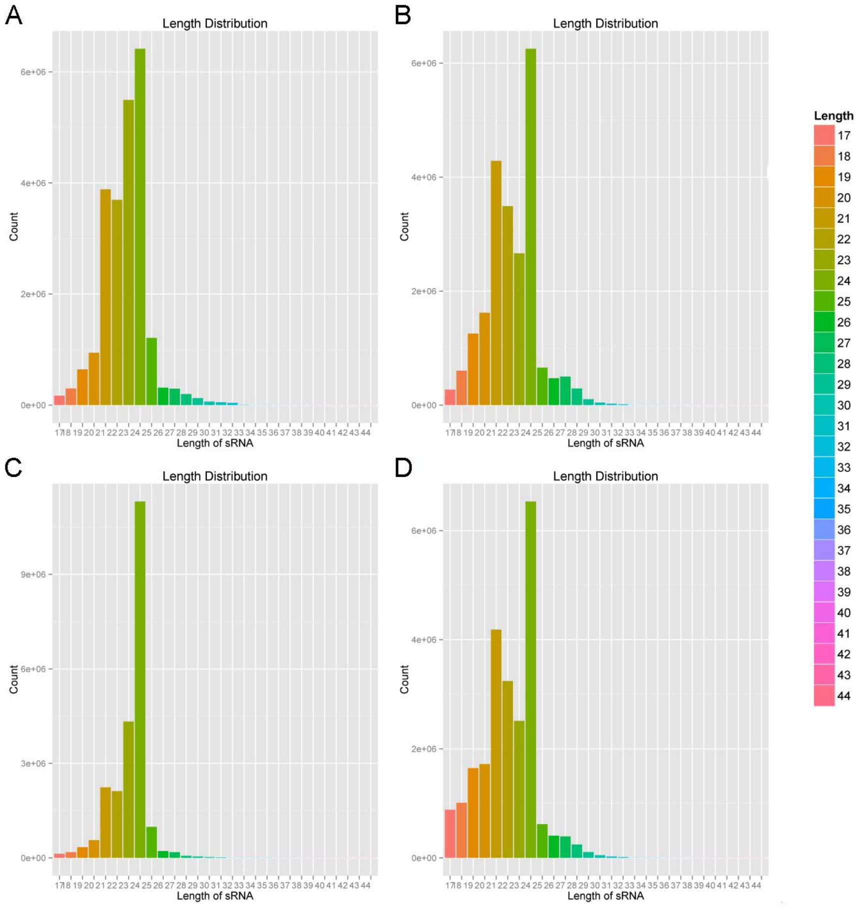

Supplementary Figure S1. Distribution of sRNAs in SS1 (A), SS2 (B), TS1 (C) and TS2 (D).

novel_mir6 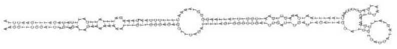

Sequence (mature) GTGGGCCGATCCGACTCGACTAGATTAGCA

Sequence (star) AATTTAATCAAAGCTGAATCGGTCTATTAA

novel_mir34 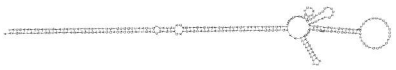

Sequence (mature) CCAAACGACGCCGATTGACCGTGC

Sequence (star) CACTGTCAATCGGCGTCGTTTGGC

novel_mir64 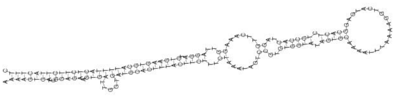

Sequence (mature) TAGGTTCAAAGGTTAGGATGTGAAGTGCA

Sequence (star) ACTTTACTTCTTTCTAAATAGTCCGTGTG

Supplementary Figure S2. Structure of predicted novel miRNA.

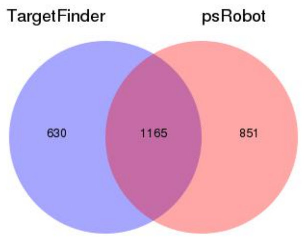

Supplementary Figure S3. A Venn diagrams showing the unique and shared regulated miRNAs predicted by TargetFinder and psRobot.

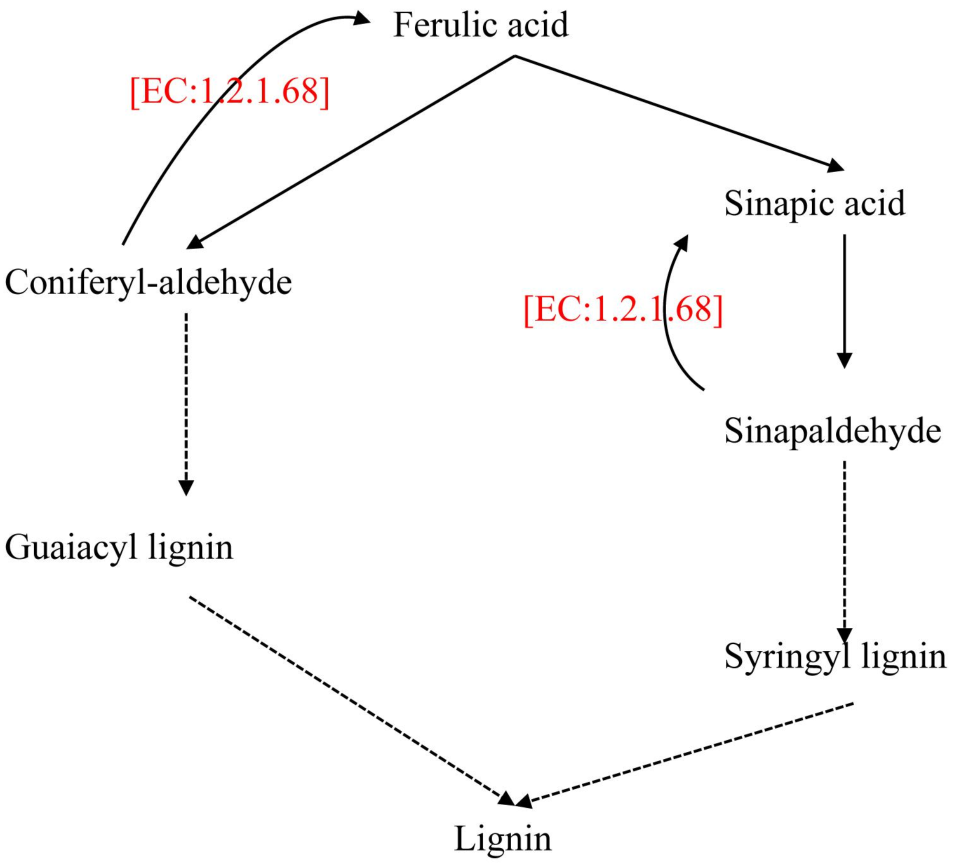

Supplementary Figure S4. Analysis of metabolic pathway for coniferyl-aldehyde dehydrogenase [EC:1.2.1.68].

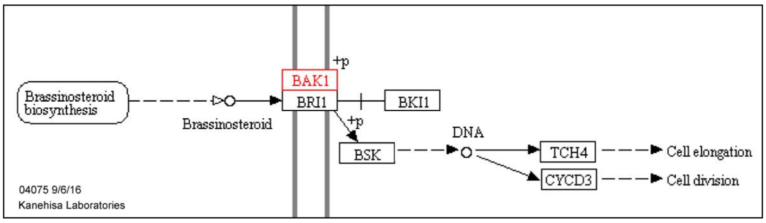

Supplementary Figure S5. Analysis of metabolic pathway for brassinosteroid insensitive 1-associated receptor kinase 1 (BAK1) with KEGG developed by Kanehisa Laboratories[74].

Supplementary Table S1. Phenotypic variation of hundred-seed weight and seed-hardness in 26 pomegranate cultivars.

ID	Hundred-seed weight(g)[a]	Seed-hardness (kg)[a]	Category
L001	6.47	4.08	hard
L002	8.09	6.49	hard
L003	4.01	1.88	soft
L004	6.84	4.63	hard
L005	8.26	7.11	hard
L006	4.28	4.07	hard
L007	6.17	5.24	hard
L008	6.19	4.34	hard
L009	5.16	6.42	hard
L010	6.72	7.16	hard
L011	4.52	4.81	hard
L012	7.8	5.49	hard
L013	4.26	3.08	hard
L014	7.73	5.3	hard
L015	5.52	6.47	hard
L016	5.21	5.86	hard
L017	6.7	5.75	hard
L018	8.61	6.68	hard
L019	6.74	6.41	hard
L020	8.43	6.91	hard
L021	6.67	6.04	hard
L022	7.71	5.37	hard
L023	8.85	6.33	hard
L024	5.89	4.41	hard
L025	8.37	6.28	hard
L026	6.8	4.76	hard

[a] mean value of trait in two environments.

Supplementary Table S2. Categorization and abundance of tags of 'Tunisa' and 'Sanbai' RNA libraries.

Sample name	Raw tag count	Clean tag count	Mapped tag	Percentage (%)	Total	Known miRNA count	Novel miRNA count
SS1	24593968	23025209	21640482	93.99	830	137	693
SS2	24362778	22362305	21359310	95.51	745	135	610
TS1	24221594	22879827	20899683	91.35	850	145	705
TS2	24222409	22523261	20423373	90.68	795	136	659

Supplementary Table S3. The miRNA family and their corresponding miRNA.

#miRNA id	Family	Mature
mdm-miR482a-5p	MIR482	AGGAATGGGCTGTTTGGGAAGA
mdm-miR482a-3p	MIR482	TTCCCAAGCCCGCCCATTCCTA
mdm-miR156a	MIR156	TGACAGAAGAGAGTGAGCAC
mdm-miR156b	MIR156	TGACAGAAGAGAGTGAGCAC
mdm-miR156c	MIR156	TGACAGAAGAGAGTGAGCAC
mdm-miR156d	MIR156	TGACAGAAGAGAGTGAGCAC
mdm-miR156e	MIR156	TGACAGAAGAGAGTGAGCAC
mdm-miR156f	MIR156	TGACAGAAGAGAGTGAGCAC
mdm-miR156g	MIR156	TGACAGAAGAGAGTGAGCAC
mdm-miR156h	MIR156	TGACAGAAGAGAGTGAGCAC
mdm-miR156i	MIR156	TGACAGAAGAGAGTGAGCAC
mdm-miR156j	MIR156	TGACAGAAGAGAGTGAGCAC
mdm-miR156k	MIR156	TGACAGAAGAGAGTGAGCAC
mdm-miR156l	MIR156	TGACAGAAGAGAGTGAGCAC
mdm-miR156m	MIR156	TGACAGAAGAGAGTGAGCAC
mdm-miR156n	MIR156	TGACAGAAGAGAGTGAGCAC
mdm-miR156o	MIR156	TGACAGAAGAGAGTGAGCAC
mdm-miR156p	MIR156	CTGACAGAAGATAGAGAGCAC
mdm-miR156q	MIR156	CTGACAGAAGATAGAGAGCAC
mdm-miR156r	MIR156	CTGACAGAAGATAGAGAGCAC
mdm-miR156s	MIR156	CTGACAGAAGATAGAGAGCAC
mdm-miR156t	MIR156	TTGACAGAAGAGAGAGAGCAC
mdm-miR156u	MIR156	TTGACAGAAGAGAGAGAGCAC
mdm-miR156v	MIR156	TTGACAGAAGAGAGAGAGCAC
mdm-miR156w	MIR156	TTGACAGAAGAGAGAGAGCAC
mdm-miR156x	MIR156	TGACAGAAGATAGAGAGCAC
mdm-miR156y	MIR156	TGACAGAAGATAGAGAGCAC
mdm-miR156z	MIR156	TGACAGAAGATAGAGAGCAC
mdm-miR156aa	MIR156	TGACAGAAGATAGAGAGCAC
mdm-miR156ab	MIR156	TTGACAGAAGATAGAGAGCAC
mdm-miR156ac	MIR156	TTGACAGAAGATAGAGAGCAC
mdm-miR156ad	MIR156	TGACAGAAGAAAGTGAGCAC
mdm-miR156ae	MIR156	TGACAGAAGAAAGTGAGCAC
mdm-miR159a	MIR159	CTTGGATTGAAGGGAGCTCC
mdm-miR159b	MIR159	CTTGGATTGAAGGGAGCTCC
mdm-miR160a	MIR160	TGCCTGGCTCCCTGTATGCCA
mdm-miR160b	MIR160	TGCCTGGCTCCCTGTATGCCA
mdm-miR160c	MIR160	TGCCTGGCTCCCTGTATGCCA
mdm-miR160d	MIR160	TGCCTGGCTCCCTGTATGCCA
mdm-miR160e	MIR160	TGCCTGGCTCCCTGTATGCCA
mdm-miR162a	MIR162_1	TCGATAAACCTCTGCATCCAG
mdm-miR162b	MIR162_1	TCGATAAACCTCTGCATCCAG

mdm-miR164a	MIR164	TGGAGAAGCAGGGCACATGCC
mdm-miR164b	MIR164	TGGAGAAGCAGGGCACGTGCA
mdm-miR164c	MIR164	TGGAGAAGCAGGGCACGTGCA
mdm-miR164d	MIR164	TGGAGAAGCAGGGCACGTGCA
mdm-miR164e	MIR164	TGGAGAAGCAGGGCACGTGCA
mdm-miR164f	MIR164	TGGAGAAGCAGGGCACGTGCA
mdm-miR166a	MIR166	TCGGACCAGGCTTCATTCCCC
mdm-miR166b	MIR166	TCGGACCAGGCTTCATTCCCC
mdm-miR166c	MIR166	TCGGACCAGGCTTCATTCCCC
mdm-miR166d	MIR166	TCGGACCAGGCTTCATTCCCC
mdm-miR166e	MIR166	TCGGACCAGGCTTCATTCCCC
mdm-miR166f	MIR166	TCGGACCAGGCTTCATTCCCC
mdm-miR166g	MIR166	TCGGACCAGGCTTCATTCCCC
mdm-miR166h	MIR166	TCGGACCAGGCTTCATTCCCC
mdm-miR166i	MIR166	TCGGACCAGGCTTCATTCCCC
mdm-miR167a	MIR167_1	AGATCATCTGGCAGTTTCACC
mdm-miR167b	MIR167_1	TGAAGCTGCCAGCATGATCTA
mdm-miR167c	MIR167_1	TGAAGCTGCCAGCATGATCTA
mdm-miR167d	MIR167_1	TGAAGCTGCCAGCATGATCTA
mdm-miR167e	MIR167_1	TGAAGCTGCCAGCATGATCTA
mdm-miR167f	MIR167_1	TGAAGCTGCCAGCATGATCTA
mdm-miR167g	MIR167_1	TGAAGCTGCCAGCATGATCTA
mdm-miR167h	MIR167_1	TGAAGCTGCCAGCATGATCTTA
mdm-miR167i	MIR167_1	TGAAGCTGCCAGCATGATCTTA
mdm-miR167j	MIR167_1	TGAAGCTGCCAGCATGATCTTA
mdm-miR168a	MIR168	TCGCTTGGTGCAGGTCGGGAA
mdm-miR168b	MIR168	TCGCTTGGTGCAGGTCGGGAA
mdm-miR169a	MIR169_2	CAGCCAAGGATGACTTGCCGG
mdm-miR169b	MIR169_1	TAGCCAAGGATGATTTGCCTGC
mdm-miR169c	MIR169_1	TAGCCAAGGATGACTTGCCCG
mdm-miR169d	MIR169_1	TAGCCAAGGATGACTTGCCCG
mdm-miR171a	MIR171_1	TTGAGCCGCGTCAATATCTCC
mdm-miR171b	MIR171_1	TTGAGCCGCGTCAATATCTCC
mdm-miR171c	MIR171_1	TGATTGAGCCGCGCCAATATC
mdm-miR171d	MIR171_1	TGATTGAGCCGCGCCAATATC
mdm-miR171e	MIR171_1	TGATTGAGCCGCGCCAATATC
mdm-miR171f	MIR171_1	TTGAGCCGTGCCAATATCACG
mdm-miR171g	MIR171_1	TGATTGAGCCGTGCCAATATC
mdm-miR171h	MIR171_1	TGATTGAGCCGTGCCAATATC
mdm-miR171i	MIR171_2	TGAGCCGAACCAATATCACTC
mdm-miR171j	MIR171_1	TTGAGCCGCGCCAATATCACT
mdm-miR171k	MIR171_1	TTGAGCCGCGCCAATATCACT
mdm-miR171l	MIR171_1	TTGAGCCGCGCCAATATCACT
mdm-miR171m	MIR171_1	TTGAGCCGTGCCAATATCACA

mdm-miR171n	MIR171_1	TTGAGCCGTGCCAATATCACA
mdm-miR172a	MIR172	AGAATCTTGATGATGCTGCA
mdm-miR172b	MIR172	AGAATCTTGATGATGCTGCA
mdm-miR172c	MIR172	AGAATCTTGATGATGCTGCA
mdm-miR172d	MIR172	AGAATCTTGATGATGCTGCAT
mdm-miR172e	MIR172	AGAATCTTGATGATGCTGCAT
mdm-miR172f	MIR172	AGAATCTTGATGATGCTGCAT
mdm-miR172g	MIR172	AGAATCTTGATGATGCTGCAT
mdm-miR172h	MIR172	AGAATCTTGATGATGCTGCAT
mdm-miR172i	MIR172	GGAATCTTGATGATGCTGCAT
mdm-miR172j	MIR172	GGAATCTTGATGATGCTGCAT
mdm-miR172k	MIR172	GGAATCTTGATGATGCTGCAT
mdm-miR172l	MIR172	GGAATCTTGATGATGCTGCAG
mdm-miR172m	MIR172	AGAATCTTGATGATGCTGCAG
mdm-miR172n	MIR172	AGAATCTTGATGATGCTGCAG
mdm-miR172o	MIR172	AGAATCTTGATGATGCTGCAG
mdm-miR319a	MIR159	TTGGACTGAAGGGAGCTCCCT
mdm-miR319b	MIR159	TTGGACTGAAGGGAGCTCCCT
mdm-miR390a	MIR390	AAGCTCAGGAGGGATAGCGCC
mdm-miR390b	MIR390	AAGCTCAGGAGGGATAGCGCC
mdm-miR390c	MIR390	AAGCTCAGGAGGGATAGCGCC
mdm-miR390d	MIR390	AAGCTCAGGAGGGATAGCGCC
mdm-miR390e	MIR390	AAGCTCAGGAGGGATAGCGCC
mdm-miR390f	MIR390	AAGCTCAGGAGGGATAGCGCC
mdm-miR393a	MIR393	TCCAAAGGGATCGCATTGATCT
mdm-miR393b	MIR393	TCCAAAGGGATCGCATTGATCT
mdm-miR393c	MIR393	TCCAAAGGGATCGCATTGATCT
mdm-miR394a	MIR394	TTGGCATTCTGTCCACCTCC
mdm-miR394b	MIR394	TTGGCATTCTGTCCACCTCC
mdm-miR395a	MIR395	CTGAAGTGTTTGGGGGAACTC
mdm-miR395b	MIR395	CTGAAGTGTTTGGGGGAACTC
mdm-miR395c	MIR395	CTGAAGTGTTTGGGGGAACTC
mdm-miR395d	MIR395	CTGAAGTGTTTGGGGGAACTC
mdm-miR395e	MIR395	CTGAAGTGTTTGGGGGAACTC
mdm-miR395f	MIR395	CTGAAGTGTTTGGGGGAACTC
mdm-miR395g	MIR395	CTGAAGTGTTTGGGGGAACTC
mdm-miR395h	MIR395	CTGAAGTGTTTGGGGGAACTC
mdm-miR395i	MIR395	CTGAAGTGTTTGGGGGAACTC
mdm-miR396a	MIR396	TTCCACAGCTTTCTTGAACAG
mdm-miR396b	MIR396	TTCCACAGCTTTCTTGAACTG
mdm-miR396c	MIR396	TTCCACAGCTTTCTTGAACTT
mdm-miR396d	MIR396	TTCCACAGCTTTCTTGAACTT
mdm-miR396e	MIR396	TTCCACAGCTTTCTTGAACTT
mdm-miR396f	MIR396	TTCCACGGCTTTCTTGAACTG

mdm-miR396g	MIR396	TTCCACGGCTTTCTTGAACTG
mdm-miR397a	MIR397	TTGAGTGCAGCGTTGATGAAA
mdm-miR397b	MIR397	TTGAGTGCAGCGTTGATGAAA
mdm-miR398a	MIR398	TGTGTTCTCAGGTCACCCCTT
mdm-miR398b	MIR398	TGTGTTCTCAGGTCGCCCCTG
mdm-miR398c	MIR398	TGTGTTCTCAGGTCGCCCCTG
mdm-miR399a	MIR399	TGCCAAAGGAGAATTGCCCTG
mdm-miR399b	MIR399	TGCCAAAGGAGAATTGCCCTG
mdm-miR399c	MIR399	TGCCAAAGGAGAATTGCCCTG
mdm-miR399d	MIR399	TGCCAAAGGAGAGTTGCCCTA
mdm-miR399e	MIR399	TGCCAAAGGAGATTTGCTCGG
mdm-miR399f	MIR399	TGCCAAAGGAGATTTGCTCGG
mdm-miR399g	MIR399	TGCCAAAGGAGATTTGCTCGG
mdm-miR399h	MIR399	TGCCAAAGGAGATTTGCTCGG
mdm-miR399i	MIR399	TGCCAAAGGAGAGTTGCCCTG
mdm-miR399j	MIR399	TGCCAAAGGAGAGTTGCCCTG
mdm-miR403a	MIR403	TTAGATTCACGCACAAACTCG
mdm-miR403b	MIR403	TTAGATTCACGCACAAACTCG
mdm-miR408a	MIR408	ATGCACTGCCTCTTCCCTGGC
mdm-miR2111a	MIR2111	TAATCTGCATCCTGAGGTTTA
mdm-miR2111b	MIR2111	TAATCTGCATCCTGAGGTTTA
mdm-miR3627a	MIR3627	TCGCAGGAGAGATGGCACTA
mdm-miR3627b	MIR3627	TCGCAGGAGAGATGGCACTA
mdm-miR3627c	MIR3627	TCGCAGGAGAGATGGCACTA
mdm-miR391	undef	TACGCAGGAGAGATGACGCCG
mdm-miR477b	MIR477	ACTCTCCCTCAAGGGCTTCGAC
mdm-miR477a	undef	ACTCTCCCTCAAGAGCTTCTC
mdm-miR482b	MIR482	TCTTTCCTATCCCTCCCATTCC
mdm-miR482c	MIR482	TCTTTCCTAACCCTCCCATTCC
mdm-miR535a	MIR535	TGACAACGAGAGAGAGCACGC
mdm-miR535b	MIR535	TGACAAGGAGAGAGAGCACGC
mdm-miR535c	MIR535	TGACAAGGAGAGAGAGCACGC
mdm-miR535d	MIR535	TGACGACGAGAGAGAGCACGC
mdm-miR827	MIR827_4	TTAGATGACCATCAACGAACA
mdm-miR828a	MIR828	TCTTGCTCAAATGAGTATTCCA
mdm-miR828b	MIR828	TCTTGCTCAAATGAGTATTCCA
mdm-miR408b	MIR408	ACAGGGAAGAGGTAGAGCATG
mdm-miR408c	MIR408	ACAGGGAAGAGGTAGAGCATG
mdm-miR408d	MIR408	ACAGGGAAGAGGTAGAGCATG
mdm-miR2118a	MIR2118_2	CTACCGATGCCACTAAGTCCCA
mdm-miR2118b	MIR2118_2	CTACCGATGCCACTAAGTCCCA
mdm-miR2118c	MIR2118_2	CTACCGATGCCACTAAGTCCCA
mdm-miR7120a	MIR7120	TGTTATATTGTCAGATTGTCA
mdm-miR7120b	MIR7120	TGTTATATTGTCAGATTGTCA

mdm-miR482d	MIR482	AATGGAAGGGTAGGAAAGAAG
mdm-miR7121a	MIR7121	TCCTCTTGGTGATCGCCCTGT
mdm-miR7121b	MIR7121	TCCTCTTGGTGATCGCCCTGT
mdm-miR7121c	MIR7121	TCCTCTTGGTGATCGCCCTGT
mdm-miR7121d	MIR7121	TCCTCTTGGTGATCGCCCTGC
mdm-miR7121e	MIR7121	TCCTCTTGGTGATCGCCCTGC
mdm-miR7121f	MIR7121	TCCTCTTGGTGATCGCCCTGC
mdm-miR7121g	MIR7121	TCCTCTTGGTGATCGCCCTGC
mdm-miR7121h	MIR7121	TCCTCTTGGTGATCGCCCTGC
mdm-miR7122a	MIR1509	TTATACAGAGAAATCACGGTCG
mdm-miR7122b	MIR1509	TTATACAGAGAAATCACGGTCG
mdm-miR7123a	MIR7123	AAGAGCGGGATGTGTAAAAGG
mdm-miR7123b	MIR7123	AAGAGCGGGATGTGTAAAAGG
mdm-miR5225c	MIR5225	TCTGTCGTGGGTGAGATGGTGC
mdm-miR159c	MIR159	GAATTCCTTCTCCTCTCCTTT
mdm-miR7124a	MIR7124	CACCAATATCAACTTTATTTG
mdm-miR7124b	MIR7124	CACCAATATCAACTTTATTTG
mdm-miR5225a	MIR5225	TCTGTCGAAGGTGAGATGGTGC
mdm-miR5225b	MIR5225	TCTGTCGAAGGTGAGATGGTGC
mdm-miR319c	MIR159	ATCCAACGAAGCAGGAGCTGA
mdm-miR7125	MIR7125	CGAACTTATTGCAACTAGCTT
mdm-miR7126	undef	AAAGTATCAAGGAGCGCAAAG
mdm-miR393d	MIR393	ATCATGCGATCCCTTCGGACG
mdm-miR393e	MIR393	ATCATGCGATCCCTTCGGACG
mdm-miR393f	MIR393	ATCATGCGATCCCTTCGGACG
mdm-miR7127a	MIR7127	ATACTCATCGAATTTGTCATA
mdm-miR7127b	MIR7127	ATACTCATCGAATTTGTCATA
mdm-miR171o	MIR171_1	TGGGATGTTGGTATGGTTCAA
mdm-miR169e	MIR169_1	TGAAGAGAAGAGCGTTGTTTGG
mdm-miR169f	MIR169_1	TGAAGAGAAGAGCGTTGTTTGG
mdm-miR7128	undef	ATCATTAACACTTAATAACGA
mdm-miR858	undef	TTCGTTGTCTGTTCGACCTGA
mdm-miR1511	MIR1511	ACCTAGCTCTGATACCATGAA

Supplementary Table S5. Differentially expressed miRNA genes in SS1_TS1 and SS2_TS2.

miRNA id	Expression(SS1)	Expression (TS1)	log2(TS1/SS1)	P value	P adj	Regulation
SS1_TS1						
novel_mir49	8.475	586.460	6.113	0.000	0.000	Up
mdm-miR171i	5.679	111.199	4.291	0.000	0.000	Up
mdm-miR156m	66.856	5.617	-3.573	0.006	0.071	Down

novel_mir 740	22.152	655.296	4.887	0.000	0.000	Up
novel_mir 610	2089.720	22025.372	3.398	0.000	0.006	Up
novel_mir 465	555.033	4884.642	3.138	0.000	0.001	Up
novel_mir 6	61.300	271.114	2.145	0.004	0.045	Up
novel_mir 129	191.750	11.662	-4.039	0.003	0.039	Down
mdm-miR 156h	1970.334	70.061	-4.814	0.000	0.004	Down
novel_mir 467	71.084	400.960	2.496	0.006	0.071	Up
novel_mir 96	2.219	83.707	5.237	0.000	0.002	Up
novel_mir 702	32.490	285.968	3.138	0.005	0.060	Up
novel_mir 286	485.324	2946.128	2.602	0.000	0.007	Up
novel_mir 523	14.629	106.836	2.868	0.007	0.077	Up
novel_mir 135	3.248	105.466	5.021	0.000	0.005	Up
novel_mir 600	242.640	1263.040	2.380	0.002	0.036	Up
novel_mir 446	12.548	188.369	3.908	0.000	0.009	Up
novel_mir 352	127.892	5.803	-4.462	0.001	0.024	Down
novel_mir 494	2.534	57.137	4.495	0.000	0.008	Up
novel_mir 731	15.957	134.495	3.075	0.006	0.071	Up
novel_mir 289	238.730	57.124	-2.063	0.001	0.024	Down
novel_mir 51	2633.009	18035.080	2.776	0.000	0.005	Up
novel_mir 122	49.594	490.433	3.306	0.001	0.017	Up
novel_mir 222	825.451	2665.783	1.691	0.001	0.017	Up
mdm-miR 166h	29121.267	97.422	-8.224	0.000	0.000	Down

mdm-miR 166e	3761.336	303.012	-3.634	0.000	0.000	Down
novel_mir 364	11.171	115.649	3.372	0.001	0.017	Up
novel_mir 457	3.156	40.152	3.669	0.008	0.079	Up
novel_mir 20	246.355	2857.864	3.536	0.000	0.000	Up
mdm-miR 156c	186.276	9.292	-4.325	0.001	0.024	Down
novel_mir 191	169.470	77.347	-1.132	0.003	0.044	Down
novel_mir 742	2958.976	8936.867	1.595	0.007	0.077	Up
mdm-miR 167j	89950.379	9048.924	-3.313	0.001	0.017	Down
mdm-miR 167e	4027.222	320.165	-3.653	0.001	0.015	Down
novel_mir 229	9.073	208.313	4.521	0.000	0.009	Up
novel_mir 251	18.994	166.343	3.131	0.002	0.032	Up
mdm-miR 172b	80.693	4.412	-4.193	0.002	0.030	Down
mdm-miR 166f	4212.219	766.593	-2.458	0.000	0.012	Down
novel_mir 754	52.507	320.457	2.610	0.000	0.001	Up
novel_mir 302	5.043	100.964	4.323	0.000	0.008	Up
novel_mir 221	321.284	1795.653	2.483	0.002	0.036	Up
novel_mir 507	419.165	1195.519	1.512	0.009	0.088	Up
mdm-miR 171g	1965.473	53.387	-5.202	0.000	0.000	Down
novel_mir 407	497.068	2819.222	2.504	0.001	0.015	Up
mdm-miR 171l	493.598	5.033	-6.616	0.000	0.000	Down
mdm-miR 395g	237.591	6.713	-5.145	0.000	0.005	Down
mdm-miR 156o	27.845	2.304	-3.595	0.010	0.097	Down

novel_mir 573	41.828	298.266	2.834	0.003	0.040	Up
mdm-miR 398b	353.977	3670.577	3.374	0.007	0.076	Up
novel_mir 651	18.242	214.442	3.555	0.001	0.020	Up
mdm-miR 171h	6605.133	916.500	-2.849	0.005	0.059	Down
novel_mir 27	299.663	650.793	1.119	0.007	0.077	Up
novel_mir 154	458.534	2097.082	2.193	0.001	0.017	Up
novel_mir 671	120.800	1745.291	3.853	0.000	0.000	Up
novel_mir 421	522.645	9461.379	4.178	0.000	0.000	Up
novel_mir 309	224.359	18.826	-3.575	0.001	0.023	Down
novel_mir 259	24.974	237.307	3.248	0.002	0.036	Up
mdm-miR 390a	122.835	2.444	-5.652	0.000	0.000	Down
novel_mir 738	27.342	159.071	2.540	0.007	0.077	Up
mdm-miR 156i	556.437	5.766	-6.593	0.000	0.000	Down
novel_mir 197	117.272	7.745	-3.920	0.001	0.023	Down
novel_mir 2	43.127	294.931	2.774	0.001	0.020	Up
novel_mir 414	13.992	109.044	2.962	0.002	0.030	Up
novel_mir 464	828.311	5841.371	2.818	0.001	0.019	Up
mdm-miR 167b	175.489	1920.847	3.452	0.001	0.024	Up
novel_mir 367	586648.611	87920.368	-2.738	0.000	0.009	Down
mdm-miR 167f	17791.458	2602.483	-2.773	0.001	0.017	Down
mdm-miR 390c	191.255	2.575	-6.215	0.000	0.000	Down
mdm-miR 156f	268.104	5.964	-5.490	0.000	0.002	Down

novel_mir 755	19.449	77.889	2.002	0.006	0.071	Up
mdm-miR 168b	1633.012	22584.726	3.790	0.001	0.016	Up
novel_mir 683	54.775	208.038	1.925	0.003	0.039	Up
novel_mir 307	877.662	2831.219	1.690	0.008	0.079	Up
novel_mir 468	13.365	233.656	4.128	0.002	0.027	Up
mdm-miR 166a	28316.160	354.528	-6.320	0.000	0.000	Down
novel_mir 53	118.201	413.689	1.807	0.003	0.040	Up
mdm-miR 160a	6860.072	430.257	-3.995	0.000	0.000	Down
novel_mir 735	3.969	89.415	4.494	0.001	0.017	Up
novel_mir 759	105.376	2552.518	4.598	0.000	0.000	Up
mdm-miR 171k	255.243	30.759	-3.053	0.007	0.077	Down
mdm-miR 164e	570.322	5414.415	3.247	0.007	0.076	Up
novel_mir 611	332.807	110.119	-1.596	0.002	0.036	Down
novel_mir 489	79037.318	312422.493	1.983	0.003	0.037	Up
novel_mir 717	282.534	24.875	-3.506	0.010	0.097	Down
novel_mir 164	1206.251	9357.395	2.956	0.000	0.000	Up
novel_mir 729	45.960	397.749	3.113	0.002	0.032	Up
mdm-miR 156v	441.699	17.251	-4.678	0.000	0.008	Down
mdm-miR 166g	1090.813	6445.301	2.563	0.001	0.017	Up
novel_mir 501	25.778	201.038	2.963	0.000	0.001	Up
mdm-miR 396c	40.309	143.540	1.832	0.005	0.057	Up
novel_mir 566	7.313	206.523	4.820	0.000	0.001	Up

novel_mir 376	11.979	107.478	3.165	0.003	0.039	Up
mdm-miR 156k	1419.719	113.584	-3.644	0.006	0.070	Down
novel_mir 13	11509.879	62782.241	2.447	0.007	0.076	Up
SS2_TS2						
novel_mir 397	11.206	111.043	3.309	0.008	0.071	Up
novel_mir 319	3.058	53.434	4.127	0.001	0.015	Up
novel_mir 740	49.059	628.390	3.679	0.001	0.011	Up
novel_mir 442	35.464	693.051	4.289	0.001	0.011	Up
novel_mir 338	174.589	2148.833	3.622	0.000	0.004	Up
mdm-miR 156h	1235.772	8.182	-7.239	0.000	0.000	Down
novel_mir 65	3.701	56.409	3.930	0.001	0.014	Up
mdm-miR 396d	105.227	722.662	2.780	0.010	0.080	Up
novel_mir 590	3.957	99.872	4.658	0.000	0.001	Up
novel_mir 561	1.946	19.894	3.354	0.009	0.076	Up
novel_mir 377	2.251	66.265	4.880	0.000	0.002	Up
novel_mir 587	3.646	106.503	4.869	0.000	0.001	Up
novel_mir 182	1.843	41.124	4.480	0.000	0.007	Up
novel_mir 99	29.022	293.603	3.339	0.000	0.000	Up
novel_mir 310	5.201	82.012	3.979	0.001	0.010	Up
novel_mir 248	35.775	245.154	2.777	0.007	0.063	Up
novel_mir 213	5.041	137.351	4.768	0.000	0.001	Up
novel_mir 581	34.606	388.397	3.488	0.000	0.003	Up
novel_mir	3.934	69.852	4.150	0.001	0.014	Up

473						
novel_mir 542	20.172	178.067	3.142	0.001	0.009	Up
novel_mir 64	4.145	47.657	3.523	0.002	0.026	Up
novel_mir 225	6.511	140.235	4.429	0.000	0.005	Up
novel_mir 187	257.981	519.914	1.011	0.010	0.080	Up
novel_mir 142	4.388	47.149	3.426	0.005	0.045	Up
novel_mir 586	54.395	222.709	2.034	0.008	0.068	Up
novel_mir 40	18.278	232.550	3.669	0.000	0.000	Up
mdm-miR 166h	352.984	126.321	-1.483	0.005	0.045	Down
novel_mir 370	4.166	54.143	3.700	0.004	0.038	Up
novel_mir 662	51.009	234.403	2.200	0.005	0.046	Up
mdm-miR 166e	21386.416	266.827	-6.325	0.000	0.000	Down
novel_mir 341	31.766	242.638	2.933	0.002	0.025	Up
novel_mir 688	4.431	168.691	5.251	0.000	0.000	Up
novel_mir 419	2.760	28.654	3.376	0.009	0.073	Up
novel_mir 608	1.711	76.560	5.483	0.000	0.000	Up
mdm-miR 167j	69954.488	14693.664	-2.251	0.012	0.095	Down
mdm-miR 167e	7182.633	121.352	-5.887	0.000	0.000	Down
novel_mir 57	61.472	421.968	2.779	0.009	0.073	Up
novel_mir 485	23.294	159.017	2.771	0.003	0.030	Up
novel_mir 71	1.909	18.341	3.264	0.011	0.089	Up
novel_mir 308	2.370	26.126	3.463	0.007	0.063	Up
novel_mir	34.977	307.637	3.137	0.001	0.018	Up

570						
novel_mir 441	3.280	39.371	3.585	0.003	0.031	Up
novel_mir 54	6.109	67.635	3.469	0.005	0.046	Up
novel_mir 477	28.829	197.369	2.775	0.001	0.010	Up
novel_mir 588	1.582	57.063	5.172	0.000	0.001	Up
novel_mir 484	2.348	111.879	5.575	0.000	0.000	Up
novel_mir 744	5.087	129.279	4.668	0.000	0.001	Up
mdm-miR 166c	11492.319	1421.808	-3.015	0.002	0.019	Down
mdm-miR 156w	573.424	31.334	-4.194	0.001	0.015	Down
novel_mir 449	3.333	66.162	4.311	0.001	0.010	Up
mdm-miR 167h	69573.813	7393.006	-3.234	0.011	0.089	Down
novel_mir 725	2.648	75.188	4.828	0.000	0.001	Up
mdm-miR 171h	3831.948	659.637	-2.538	0.000	0.003	Down
novel_mir 504	9.663	131.267	3.764	0.001	0.015	Up
novel_mir 753	16.904	134.912	2.997	0.011	0.089	Up
novel_mir 593	99393.592	34891.971	-1.510	0.011	0.089	Down
novel_mir 707	3.532	56.494	4.000	0.002	0.020	Up
mdm-miR 390e	302.381	10.046	-4.912	0.000	0.003	Down
novel_mir 711	4.559	90.219	4.306	0.000	0.005	Up
novel_mir 536	2.867	44.219	3.947	0.001	0.011	Up
novel_mir 161	3.800	88.222	4.537	0.000	0.001	Up
novel_mir 578	87.176	440.626	2.338	0.000	0.007	Up
novel_mir	1.720	75.221	5.450	0.000	0.000	Up

335						
mdm-miR 156l	141.528	5.968	-4.568	0.000	0.008	Down
novel_mir 258	2.554	49.906	4.288	0.001	0.010	Up
mdm-miR 156s	8.396	0.298	-4.817	0.000	0.006	Down
novel_mir 757	5.891	191.306	5.021	0.000	0.001	Up
novel_mir 280	30.388	210.869	2.795	0.004	0.036	Up
novel_mir 11	13.277	0.354	-5.231	0.000	0.002	Down
mdm-miR 156ac	100.882	5.978	-4.077	0.000	0.002	Down
novel_mir 552	6.430	175.686	4.772	0.000	0.001	Up
mdm-miR 164d	80.883	1248.349	3.948	0.000	0.009	Up
novel_mir 365	27.691	351.252	3.665	0.002	0.022	Up
mdm-miR 167b	15038.025	474.617	-4.986	0.000	0.002	Down
novel_mir 316	1.304	27.847	4.417	0.000	0.007	Up
novel_mir 479	2.757	66.459	4.591	0.000	0.001	Up
novel_mir 723	41.119	204.848	2.317	0.003	0.029	Up
novel_mir 80	363.509	2233.364	2.619	0.009	0.073	Up
novel_mir 162	6.408	76.956	3.586	0.003	0.029	Up
novel_mir 91	1.377	45.238	5.038	0.000	0.001	Up
novel_mir 537	1125.664	5062.264	2.169	0.008	0.069	Up
mdm-miR 156j	1630.348	6.100	-8.062	0.000	0.000	Down
novel_mir 558	14.174	122.635	3.113	0.002	0.026	Up
novel_mir 623	4.406	87.971	4.320	0.000	0.004	Up
novel_mir	1814.512	510.171	-1.831	0.002	0.024	Down

403						
novel_mir 240	860.122	116.537	-2.884	0.000	0.008	Down
novel_mir 132	510.103	64.724	-2.978	0.000	0.000	Down
novel_mir 7	13.668	298.774	4.450	0.000	0.008	Up
novel_mir 468	16.437	230.584	3.810	0.002	0.026	Up
novel_mir 436	78.074	309.647	1.988	0.006	0.055	Up
mdm-miR 166i	9809.451	200.121	-5.615	0.000	0.000	Down
mdm-miR 160a	1972.696	473.778	-2.058	0.000	0.008	Down
novel_mir 36	2.526	44.882	4.151	0.001	0.014	Up
novel_mir 389	6.344	73.826	3.541	0.004	0.042	Up
novel_mir 620	37.992	290.008	2.932	0.003	0.031	Up
novel_mir 759	273.524	2384.370	3.124	0.004	0.037	Up
novel_mir 147	5.083	104.706	4.365	0.000	0.009	Up
novel_mir 63	6.357	55.622	3.129	0.008	0.073	Up
mdm-miR 167i	65157.466	12258.391	-2.410	0.001	0.011	Down
mdm-miR 168a	61279.706	7479.719	-3.034	0.003	0.029	Down
novel_mir 677	3.536	101.240	4.839	0.000	0.001	Up
novel_mir 462	2419.757	286.012	-3.081	0.001	0.010	Down
novel_mir 453	18.934	169.169	3.159	0.006	0.056	Up
mdm-miR 166g	1091.716	6501.505	2.574	0.001	0.019	Up
novel_mir 511	104.365	294.171	1.495	0.003	0.032	Up
novel_mir 469	27.002	309.116	3.517	0.000	0.008	Up
mdm-miR	1123.329	116.934	-3.264	0.002	0.025	Down

396c						
novel_mir375	131.170	400.896	1.612	0.006	0.056	Up
novel_mir560	58.479	367.815	2.653	0.000	0.007	Up
novel_mir503	1223.338	138.503	-3.143	0.000	0.003	Down
novel_mir682	5.849	54.357	3.216	0.007	0.061	Up
novel_mir332	5813.707	1673.975	-1.796	0.010	0.080	Down
mdm-miR164f	3297.987	232.504	-3.826	0.001	0.013	Down
novel_mir349	5.671	80.886	3.834	0.001	0.015	Up
mdm-miR156k	58.857	3.391	-4.118	0.000	0.000	Down
novel_mir189	19.204	175.142	3.189	0.000	0.007	Up
novel_mir177	2.413	36.733	3.928	0.002	0.023	Up
novel_mir346	6.487	119.082	4.198	0.000	0.005	Up
mdm-miR395d	81.663	4.766	-4.099	0.001	0.018	Down
novel_mir758	4.274	54.488	3.672	0.004	0.041	Up

Supplementary Table S6. Target genes predicted for differentially expressed miRNAs.

miRNA id	Target gene id
SS1_TS1	
novel_mir49	Gglean002115.1
novel_mir49	Gglean002110.1
mdm-miR171i	Gglean026929.1
mdm-miR171i	Gglean025172.1
mdm-miR171i	Gglean015276.1
mdm-miR171i	Gglean007369.1
mdm-miR171i	Gglean029630.1
mdm-miR171i	Gglean015279.1
mdm-miR171i	Gglean015277.1
mdm-miR156m	Gglean028488.1
mdm-miR156m	Gglean028306.1
mdm-miR156m	Gglean004741.1
mdm-miR156m	Gglean029526.1

mdm-miR156m	Gglean005913.1
mdm-miR156m	Gglean005740.1
mdm-miR156m	Gglean016490.1
mdm-miR156m	Gglean009481.1
mdm-miR156m	Gglean016197.1
mdm-miR156m	Gglean000364.1
mdm-miR156m	Gglean005554.1
mdm-miR156m	Gglean006983.1
mdm-miR156m	Gglean007788.1
mdm-miR156m	Gglean001912.1
mdm-miR156m	Gglean008959.1
mdm-miR156m	Gglean017894.1
mdm-miR156m	Gglean016208.1
novel_mir465	Gglean005528.1
mdm-miR156h	Gglean028488.1
mdm-miR156h	Gglean028306.1
mdm-miR156h	Gglean004741.1
mdm-miR156h	Gglean029526.1
mdm-miR156h	Gglean005740.1
mdm-miR156h	Gglean016490.1
mdm-miR156h	Gglean009481.1
mdm-miR156h	Gglean016197.1
mdm-miR156h	Gglean000364.1
mdm-miR156h	Gglean005554.1
mdm-miR156h	Gglean006983.1
mdm-miR156h	Gglean000847.1
mdm-miR156h	Gglean007788.1
mdm-miR156h	Gglean001912.1
mdm-miR156h	Gglean017894.1
mdm-miR156h	Gglean016208.1
novel_mir467	Gglean015850.1
novel_mir467	Gglean024339.1
novel_mir467	Gglean027182.1
novel_mir600	Gglean024108.1
novel_mir600	Gglean026004.1
novel_mir600	Gglean022320.1
novel_mir600	Gglean019524.1
novel_mir600	Gglean017007.1
novel_mir600	Gglean025514.1
novel_mir222	Gglean023104.1
novel_mir222	Gglean014013.1
novel_mir222	Gglean002236.1
novel_mir222	Gglean021963.1
novel_mir222	Gglean004883.1

novel_mir222	Gglean016779.1
novel_mir222	Gglean015012.1
novel_mir222	Gglean017955.1
novel_mir222	Gglean016215.1
novel_mir222	Gglean006526.1
mdm-miR166h	Gglean031286.1
mdm-miR166h	Gglean027183.1
mdm-miR166h	Gglean013966.1
mdm-miR166h	Gglean013488.1
mdm-miR166e	Gglean031286.1
mdm-miR166e	Gglean027183.1
mdm-miR166e	Gglean012177.1
mdm-miR166e	Gglean013966.1
mdm-miR166e	Gglean013488.1
novel_mir364	Gglean010205.1
novel_mir364	Gglean024484.1
novel_mir364	Gglean006249.1
mdm-miR156c	Gglean028488.1
mdm-miR156c	Gglean028306.1
mdm-miR156c	Gglean004741.1
mdm-miR156c	Gglean029526.1
mdm-miR156c	Gglean005913.1
mdm-miR156c	Gglean005740.1
mdm-miR156c	Gglean016490.1
mdm-miR156c	Gglean009481.1
mdm-miR156c	Gglean016197.1
mdm-miR156c	Gglean000364.1
mdm-miR156c	Gglean005554.1
mdm-miR156c	Gglean006983.1
mdm-miR156c	Gglean007788.1
mdm-miR156c	Gglean001912.1
mdm-miR156c	Gglean008959.1
mdm-miR156c	Gglean017894.1
mdm-miR156c	Gglean016208.1
mdm-miR167j	Gglean024889.1
mdm-miR167j	Gglean022780.1
mdm-miR167e	Gglean012764.1
mdm-miR167e	Gglean024889.1
mdm-miR167e	Gglean004930.1
mdm-miR167e	Gglean011855.1
mdm-miR167e	Gglean029426.1
mdm-miR167e	Gglean005024.1
mdm-miR167e	Gglean027622.1
novel_mir229	Gglean014246.1

novel_mir229	Gglean005543.1
novel_mir251	Gglean008005.1
mdm-miR172b	Gglean026155.1
mdm-miR172b	Gglean005692.1
mdm-miR172b	Gglean015520.1
mdm-miR172b	Gglean003110.1
mdm-miR172b	Gglean025204.1
mdm-miR172b	Gglean028555.1
mdm-miR172b	Gglean031260.1
mdm-miR172b	Gglean011686.1
mdm-miR172b	Gglean024299.1
mdm-miR172b	Gglean003632.1
mdm-miR172b	Gglean015192.1
mdm-miR172b	Gglean026282.1
mdm-miR172b	Gglean025892.1
mdm-miR172b	Gglean021038.1
mdm-miR172b	Gglean024792.1
mdm-miR172b	Gglean008425.1
mdm-miR172b	Gglean013091.1
mdm-miR172b	Gglean027909.1
mdm-miR172b	Gglean021449.1
mdm-miR172b	Gglean012497.1
mdm-miR172b	Gglean026243.1
mdm-miR172b	Gglean010833.1
mdm-miR172b	Gglean021957.1
mdm-miR172b	Gglean026848.1
mdm-miR172b	Gglean022370.1
mdm-miR172b	Gglean027146.1
mdm-miR172b	Gglean017396.1
mdm-miR172b	Gglean026849.1
mdm-miR172b	Gglean029466.1
mdm-miR172b	Gglean030416.1
mdm-miR172b	Gglean020028.1
mdm-miR172b	Gglean014137.1
mdm-miR172b	Gglean019483.1
mdm-miR172b	Gglean030854.1
mdm-miR172b	Gglean025466.1
mdm-miR172b	Gglean026000.1
mdm-miR172b	Gglean000051.1
mdm-miR166f	Gglean031286.1
mdm-miR166f	Gglean027183.1
mdm-miR166f	Gglean012177.1
mdm-miR166f	Gglean013966.1
mdm-miR166f	Gglean013488.1

mdm-miR171g	Gglean025172.1
mdm-miR171g	Gglean017038.1
mdm-miR171g	Gglean006729.1
mdm-miR171l	Gglean025172.1
mdm-miR171l	Gglean006729.1
mdm-miR171l	Gglean007369.1
mdm-miR171l	Gglean030916.1
mdm-miR171l	Gglean029440.1
mdm-miR395g	Gglean006692.1
mdm-miR395g	Gglean005238.1
mdm-miR395g	Gglean030038.1
mdm-miR395g	Gglean022778.1
mdm-miR395g	Gglean015303.1
mdm-miR395g	Gglean008746.1
mdm-miR156o	Gglean028488.1
mdm-miR156o	Gglean028306.1
mdm-miR156o	Gglean004741.1
mdm-miR156o	Gglean029526.1
mdm-miR156o	Gglean005913.1
mdm-miR156o	Gglean005740.1
mdm-miR156o	Gglean016490.1
mdm-miR156o	Gglean009481.1
mdm-miR156o	Gglean016197.1
mdm-miR156o	Gglean000364.1
mdm-miR156o	Gglean005554.1
mdm-miR156o	Gglean006983.1
mdm-miR156o	Gglean000847.1
mdm-miR156o	Gglean007788.1
mdm-miR156o	Gglean001912.1
mdm-miR156o	Gglean008959.1
mdm-miR156o	Gglean017894.1
mdm-miR156o	Gglean016208.1
mdm-miR398b	Gglean021111.1
mdm-miR398b	Gglean005184.1
mdm-miR398b	Gglean005399.1
mdm-miR171h	Gglean025172.1
mdm-miR171h	Gglean017038.1
mdm-miR171h	Gglean006729.1
novel_mir27	Gglean003050.1
novel_mir154	Gglean001619.1
novel_mir671	Gglean007251.1
novel_mir671	Gglean023976.1
novel_mir671	Gglean001930.1
novel_mir671	Gglean018106.1

novel_mir671	Gglean000795.1
novel_mir671	Gglean001373.1
novel_mir671	Gglean023985.1
novel_mir671	Gglean030172.1
novel_mir671	Gglean019633.1
novel_mir671	Gglean026208.1
novel_mir671	Gglean004793.1
novel_mir671	Gglean028372.1
novel_mir671	Gglean019038.1
novel_mir671	Gglean001064.1
novel_mir671	Gglean021739.1
novel_mir671	Gglean026964.1
novel_mir671	Gglean008120.1
novel_mir671	Gglean010791.1
novel_mir671	Gglean026774.1
novel_mir671	Gglean027535.1
novel_mir671	Gglean004424.1
novel_mir671	Gglean001374.1
novel_mir671	Gglean001138.1
novel_mir671	Gglean013094.1
novel_mir671	Gglean025059.1
novel_mir671	Gglean030732.1
novel_mir671	Gglean030433.1
novel_mir671	Gglean005089.1
novel_mir671	Gglean011852.1
novel_mir671	Gglean008587.1
novel_mir671	Gglean005422.1
novel_mir421	Gglean028274.1
novel_mir421	Gglean025663.1
novel_mir421	Gglean000219.1
novel_mir421	Gglean030041.1
novel_mir421	Gglean021204.1
mdm-miR390a	Gglean025452.1
mdm-miR390a	Gglean000925.1
mdm-miR390a	Gglean003665.1
mdm-miR390a	Gglean015516.1
mdm-miR390a	Gglean015873.1
mdm-miR390a	Gglean029810.1
mdm-miR390a	Gglean022261.1
mdm-miR390a	Gglean004620.1
mdm-miR390a	Gglean018883.1
mdm-miR390a	Gglean012251.1
mdm-miR390a	Gglean016147.1
novel_mir738	Gglean027156.1

novel_mir738	Gglean025642.1
novel_mir738	Gglean017975.1
mdm-miR156i	Gglean028488.1
mdm-miR156i	Gglean028306.1
mdm-miR156i	Gglean004741.1
mdm-miR156i	Gglean029526.1
mdm-miR156i	Gglean005913.1
mdm-miR156i	Gglean005740.1
mdm-miR156i	Gglean016490.1
mdm-miR156i	Gglean009481.1
mdm-miR156i	Gglean016197.1
mdm-miR156i	Gglean000364.1
mdm-miR156i	Gglean005554.1
mdm-miR156i	Gglean000847.1
mdm-miR156i	Gglean007788.1
mdm-miR156i	Gglean001912.1
mdm-miR156i	Gglean008959.1
mdm-miR156i	Gglean017894.1
mdm-miR156i	Gglean016208.1
novel_mir2	Gglean029881.1
novel_mir2	Gglean013741.1
mdm-miR167b	Gglean012764.1
mdm-miR167b	Gglean024889.1
mdm-miR167b	Gglean004930.1
mdm-miR167b	Gglean011855.1
mdm-miR167b	Gglean005024.1
mdm-miR167b	Gglean029426.1
mdm-miR167b	Gglean027622.1
novel_mir367	Gglean018231.1
novel_mir367	Gglean009130.1
novel_mir367	Gglean018236.1
novel_mir367	Gglean026867.1
novel_mir367	Gglean009129.1
novel_mir367	Gglean023388.1
mdm-miR167f	Gglean012764.1
mdm-miR167f	Gglean024889.1
mdm-miR167f	Gglean004930.1
mdm-miR167f	Gglean011855.1
mdm-miR167f	Gglean027622.1
mdm-miR167f	Gglean005024.1
mdm-miR167f	Gglean029426.1
mdm-miR390c	Gglean025452.1
mdm-miR390c	Gglean000925.1
mdm-miR390c	Gglean003665.1

mdm-miR390c	Gglean015516.1
mdm-miR390c	Gglean029810.1
mdm-miR390c	Gglean015873.1
mdm-miR390c	Gglean022261.1
mdm-miR390c	Gglean004620.1
mdm-miR390c	Gglean018883.1
mdm-miR390c	Gglean012251.1
mdm-miR390c	Gglean016147.1
mdm-miR156f	Gglean028488.1
mdm-miR156f	Gglean028306.1
mdm-miR156f	Gglean004741.1
mdm-miR156f	Gglean029526.1
mdm-miR156f	Gglean005913.1
mdm-miR156f	Gglean016490.1
mdm-miR156f	Gglean009481.1
mdm-miR156f	Gglean016197.1
mdm-miR156f	Gglean000364.1
mdm-miR156f	Gglean005554.1
mdm-miR156f	Gglean000847.1
mdm-miR156f	Gglean006983.1
mdm-miR156f	Gglean007788.1
mdm-miR156f	Gglean001912.1
mdm-miR156f	Gglean008959.1
mdm-miR156f	Gglean017894.1
mdm-miR156f	Gglean016208.1
mdm-miR168b	Gglean028715.1
mdm-miR168b	Gglean028716.1
novel_mir468	Gglean000924.1
novel_mir468	Gglean025588.1
novel_mir468	Gglean016418.1
novel_mir468	Gglean022759.1
novel_mir468	Gglean013938.1
novel_mir468	Gglean002368.1
novel_mir468	Gglean014421.1
novel_mir468	Gglean005380.1
novel_mir468	Gglean017857.1
mdm-miR166a	Gglean031286.1
mdm-miR166a	Gglean027183.1
mdm-miR166a	Gglean012177.1
mdm-miR166a	Gglean013966.1
mdm-miR166a	Gglean013488.1
mdm-miR160a	Gglean025700.1
mdm-miR160a	Gglean003992.1
mdm-miR160a	Gglean026255.1

mdm-miR160a	Gglean000179.1
mdm-miR160a	Gglean024332.1
mdm-miR171k	Gglean025172.1
mdm-miR171k	Gglean006729.1
mdm-miR171k	Gglean007369.1
mdm-miR171k	Gglean030916.1
mdm-miR171k	Gglean029440.1
mdm-miR164e	Gglean022642.1
mdm-miR164e	Gglean005008.1
mdm-miR164e	Gglean003381.1
mdm-miR164e	Gglean001478.1
mdm-miR164e	Gglean007434.1
mdm-miR164e	Gglean016084.1
mdm-miR164e	Gglean025995.1
mdm-miR164e	Gglean000139.1
mdm-miR164e	Gglean027714.1
mdm-miR164e	Gglean031067.1
mdm-miR164e	Gglean003444.1
mdm-miR164e	Gglean026964.1
novel_mir611	Gglean023296.1
novel_mir717	Gglean022344.1
novel_mir717	Gglean013148.1
novel_mir717	Gglean003916.1
novel_mir717	Gglean013578.1
novel_mir717	Gglean024339.1
novel_mir717	Gglean030793.1
mdm-miR156v	Gglean028488.1
mdm-miR156v	Gglean028306.1
mdm-miR156v	Gglean004741.1
mdm-miR156v	Gglean006999.1
mdm-miR156v	Gglean001912.1
mdm-miR156v	Gglean015358.1
mdm-miR156v	Gglean026384.1
mdm-miR156v	Gglean016197.1
mdm-miR156v	Gglean016208.1
mdm-miR156v	Gglean006983.1
mdm-miR156v	Gglean000847.1
mdm-miR166g	Gglean031286.1
mdm-miR166g	Gglean027183.1
mdm-miR166g	Gglean012177.1
mdm-miR166g	Gglean013966.1
mdm-miR166g	Gglean013488.1
mdm-miR396c	Gglean029554.1
mdm-miR396c	Gglean000122.1

mdm-miR396c	Gglean030172.1
mdm-miR396c	Gglean011692.1
mdm-miR396c	Gglean017067.1
mdm-miR396c	Gglean002260.1
mdm-miR396c	Gglean012701.1
mdm-miR396c	Gglean026508.1
mdm-miR396c	Gglean029171.1
mdm-miR396c	Gglean015340.1
mdm-miR396c	Gglean006981.1
mdm-miR396c	Gglean013328.1
mdm-miR396c	Gglean024774.1
mdm-miR396c	Gglean012175.1
mdm-miR396c	Gglean023132.1
mdm-miR396c	Gglean002322.1
mdm-miR396c	Gglean025329.1
mdm-miR396c	Gglean028260.1
novel_mir566	Gglean005510.1
novel_mir566	Gglean027274.1
novel_mir566	Gglean015850.1
novel_mir566	Gglean021934.1
novel_mir566	Gglean021229.1
novel_mir566	Gglean013148.1
novel_mir566	Gglean018517.1
novel_mir566	Gglean000351.1
novel_mir566	Gglean027182.1
novel_mir376	Gglean005264.1
mdm-miR156k	Gglean028488.1
mdm-miR156k	Gglean028306.1
mdm-miR156k	Gglean004741.1
mdm-miR156k	Gglean029526.1
mdm-miR156k	Gglean005913.1
mdm-miR156k	Gglean005740.1
mdm-miR156k	Gglean016490.1
mdm-miR156k	Gglean009481.1
mdm-miR156k	Gglean016197.1
mdm-miR156k	Gglean000364.1
mdm-miR156k	Gglean005554.1
mdm-miR156k	Gglean000847.1
mdm-miR156k	Gglean007788.1
mdm-miR156k	Gglean001912.1
mdm-miR156k	Gglean008959.1
mdm-miR156k	Gglean017894.1
mdm-miR156k	Gglean016208.1

SS2_TS2

novel_mir338	Gglean017161.1
novel_mir338	Gglean006371.1
mdm-miR156h	Gglean028488.1
mdm-miR156h	Gglean028306.1
mdm-miR156h	Gglean004741.1
mdm-miR156h	Gglean029526.1
mdm-miR156h	Gglean005740.1
mdm-miR156h	Gglean016490.1
mdm-miR156h	Gglean009481.1
mdm-miR156h	Gglean016197.1
mdm-miR156h	Gglean000364.1
mdm-miR156h	Gglean005554.1
mdm-miR156h	Gglean006983.1
mdm-miR156h	Gglean000847.1
mdm-miR156h	Gglean007788.1
mdm-miR156h	Gglean001912.1
mdm-miR156h	Gglean017894.1
mdm-miR156h	Gglean016208.1
mdm-miR396d	Gglean029554.1
mdm-miR396d	Gglean000122.1
mdm-miR396d	Gglean030172.1
mdm-miR396d	Gglean011692.1
mdm-miR396d	Gglean017067.1
mdm-miR396d	Gglean002260.1
mdm-miR396d	Gglean029171.1
mdm-miR396d	Gglean026508.1
mdm-miR396d	Gglean015340.1
mdm-miR396d	Gglean006981.1
mdm-miR396d	Gglean013328.1
mdm-miR396d	Gglean024774.1
mdm-miR396d	Gglean012175.1
mdm-miR396d	Gglean023132.1
mdm-miR396d	Gglean025329.1
mdm-miR396d	Gglean028260.1
novel_mir225	Gglean027862.1
mdm-miR166h	Gglean031286.1
mdm-miR166h	Gglean027183.1
mdm-miR166h	Gglean013966.1
mdm-miR166h	Gglean013488.1
novel_mir370	Gglean007745.1
novel_mir370	Gglean017934.1
novel_mir662	Gglean029606.1
mdm-miR166e	Gglean031286.1
mdm-miR166e	Gglean027183.1

mdm-miR166e	Gglean012177.1
mdm-miR166e	Gglean013966.1
mdm-miR166e	Gglean013488.1
novel_mir608	Gglean019246.1
novel_mir608	Gglean007215.1
novel_mir608	Gglean028657.1
novel_mir608	Gglean026765.1
novel_mir608	Gglean000622.1
novel_mir608	Gglean003735.1
novel_mir608	Gglean011838.1
mdm-miR167j	Gglean024889.1
mdm-miR167j	Gglean022780.1
mdm-miR167e	Gglean012764.1
mdm-miR167e	Gglean024889.1
mdm-miR167e	Gglean004930.1
mdm-miR167e	Gglean011855.1
mdm-miR167e	Gglean029426.1
mdm-miR167e	Gglean005024.1
mdm-miR167e	Gglean027622.1
novel_mir57	Gglean005623.1
novel_mir57	Gglean028948.1
novel_mir71	Gglean003943.1
novel_mir71	Gglean019081.1
novel_mir71	Gglean018771.1
novel_mir308	Gglean020252.1
novel_mir744	Gglean003829.1
novel_mir744	Gglean020429.1
novel_mir744	Gglean029338.1
novel_mir744	Gglean003814.1
novel_mir744	Gglean003490.1
novel_mir744	Gglean019566.1
novel_mir744	Gglean007846.1
novel_mir744	Gglean026280.1
novel_mir744	Gglean007878.1
novel_mir744	Gglean009519.1
novel_mir744	Gglean010681.1
novel_mir744	Gglean009266.1
novel_mir744	Gglean024152.1
novel_mir744	Gglean002036.1
novel_mir744	Gglean002649.1
novel_mir744	Gglean015202.1
novel_mir744	Gglean026028.1
novel_mir744	Gglean024317.1
novel_mir744	Gglean027546.1

novel_mir744	Gglean021390.1
novel_mir744	Gglean015742.1
novel_mir744	Gglean014227.1
novel_mir744	Gglean027784.1
novel_mir744	Gglean029291.1
novel_mir744	Gglean029008.1
novel_mir744	Gglean002322.1
novel_mir744	Gglean020055.1
novel_mir744	Gglean002537.1
mdm-miR166c	Gglean031286.1
mdm-miR166c	Gglean027183.1
mdm-miR166c	Gglean012177.1
mdm-miR166c	Gglean013966.1
mdm-miR166c	Gglean013488.1
mdm-miR156w	Gglean028488.1
mdm-miR156w	Gglean028306.1
mdm-miR156w	Gglean004741.1
mdm-miR156w	Gglean006999.1
mdm-miR156w	Gglean001912.1
mdm-miR156w	Gglean015358.1
mdm-miR156w	Gglean026384.1
mdm-miR156w	Gglean016197.1
mdm-miR156w	Gglean016208.1
mdm-miR156w	Gglean000847.1
mdm-miR156w	Gglean006983.1
novel_mir449	Gglean008860.1
mdm-miR167h	Gglean024889.1
mdm-miR167h	Gglean022780.1
novel_mir725	Gglean017451.1
novel_mir725	Gglean019677.1
novel_mir725	Gglean020906.1
novel_mir725	Gglean024157.1
mdm-miR171h	Gglean025172.1
mdm-miR171h	Gglean017038.1
mdm-miR171h	Gglean006729.1
mdm-miR390e	Gglean025452.1
mdm-miR390e	Gglean000925.1
mdm-miR390e	Gglean003665.1
mdm-miR390e	Gglean015516.1
mdm-miR390e	Gglean015873.1
mdm-miR390e	Gglean029810.1
mdm-miR390e	Gglean022261.1
mdm-miR390e	Gglean004620.1
mdm-miR390e	Gglean018883.1

mdm-miR390e	Gglean012251.1
mdm-miR390e	Gglean016147.1
mdm-miR156l	Gglean028488.1
mdm-miR156l	Gglean028306.1
mdm-miR156l	Gglean004741.1
mdm-miR156l	Gglean029526.1
mdm-miR156l	Gglean005913.1
mdm-miR156l	Gglean005740.1
mdm-miR156l	Gglean016490.1
mdm-miR156l	Gglean009481.1
mdm-miR156l	Gglean016197.1
mdm-miR156l	Gglean000364.1
mdm-miR156l	Gglean005554.1
mdm-miR156l	Gglean000847.1
mdm-miR156l	Gglean006983.1
mdm-miR156l	Gglean007788.1
mdm-miR156l	Gglean001912.1
mdm-miR156l	Gglean008959.1
mdm-miR156l	Gglean017894.1
mdm-miR156l	Gglean016208.1
mdm-miR156s	Gglean028488.1
mdm-miR156s	Gglean028306.1
mdm-miR156s	Gglean004741.1
mdm-miR156s	Gglean028908.1
mdm-miR156s	Gglean020280.1
mdm-miR156s	Gglean001913.1
mdm-miR156s	Gglean016197.1
mdm-miR156s	Gglean016208.1
mdm-miR156s	Gglean009943.1
novel_mir757	Gglean000171.1
novel_mir757	Gglean001497.1
novel_mir757	Gglean020474.1
novel_mir11	Gglean000349.1
novel_mir11	Gglean002650.1
mdm-miR156ac	Gglean028488.1
mdm-miR156ac	Gglean010399.1
mdm-miR156ac	Gglean028306.1
mdm-miR156ac	Gglean004741.1
mdm-miR156ac	Gglean020280.1
mdm-miR156ac	Gglean012943.1
mdm-miR156ac	Gglean022143.1
mdm-miR156ac	Gglean001056.1
mdm-miR156ac	Gglean005740.1
mdm-miR156ac	Gglean016197.1

mdm-miR156ac	Gglean016208.1
mdm-miR164d	Gglean022642.1
mdm-miR164d	Gglean005008.1
mdm-miR164d	Gglean003381.1
mdm-miR164d	Gglean007834.1
mdm-miR164d	Gglean007434.1
mdm-miR164d	Gglean016084.1
mdm-miR164d	Gglean025995.1
mdm-miR164d	Gglean000139.1
mdm-miR164d	Gglean027714.1
mdm-miR164d	Gglean031067.1
mdm-miR164d	Gglean003444.1
mdm-miR164d	Gglean026964.1
mdm-miR167b	Gglean012764.1
mdm-miR167b	Gglean024889.1
mdm-miR167b	Gglean004930.1
mdm-miR167b	Gglean011855.1
mdm-miR167b	Gglean005024.1
mdm-miR167b	Gglean029426.1
mdm-miR167b	Gglean027622.1
novel_mir91	Gglean030408.1
novel_mir91	Gglean000862.1
mdm-miR156j	Gglean028488.1
mdm-miR156j	Gglean028306.1
mdm-miR156j	Gglean004741.1
mdm-miR156j	Gglean007788.1
mdm-miR156j	Gglean029526.1
mdm-miR156j	Gglean001912.1
mdm-miR156j	Gglean016490.1
mdm-miR156j	Gglean008959.1
mdm-miR156j	Gglean009481.1
mdm-miR156j	Gglean016197.1
mdm-miR156j	Gglean000364.1
mdm-miR156j	Gglean016208.1
mdm-miR156j	Gglean005554.1
mdm-miR156j	Gglean006983.1
mdm-miR156j	Gglean000847.1
novel_mir403	Gglean017702.1
novel_mir468	Gglean000924.1
novel_mir468	Gglean025588.1
novel_mir468	Gglean016418.1
novel_mir468	Gglean022759.1
novel_mir468	Gglean013938.1
novel_mir468	Gglean002368.1

novel_mir468	Gglean014421.1
novel_mir468	Gglean005380.1
novel_mir468	Gglean017857.1
mdm-miR166i	Gglean031286.1
mdm-miR166i	Gglean027183.1
mdm-miR166i	Gglean012177.1
mdm-miR166i	Gglean013966.1
mdm-miR166i	Gglean013488.1
mdm-miR160a	Gglean025700.1
mdm-miR160a	Gglean003992.1
mdm-miR160a	Gglean026255.1
mdm-miR160a	Gglean000179.1
mdm-miR160a	Gglean024332.1
novel_mir389	Gglean025816.1
novel_mir389	Gglean007514.1
novel_mir389	Gglean013072.1
novel_mir389	Gglean028460.1
novel_mir389	Gglean011944.1
mdm-miR167i	Gglean024889.1
mdm-miR167i	Gglean022780.1
mdm-miR168a	Gglean028715.1
mdm-miR168a	Gglean028716.1
novel_mir677	Gglean017077.1
novel_mir677	Gglean029934.1
novel_mir677	Gglean007200.1
novel_mir677	Gglean004899.1
novel_mir677	Gglean023389.1
novel_mir677	Gglean008383.1
novel_mir677	Gglean003927.1
novel_mir677	Gglean010080.1
novel_mir677	Gglean006141.1
mdm-miR166g	Gglean031286.1
mdm-miR166g	Gglean027183.1
mdm-miR166g	Gglean012177.1
mdm-miR166g	Gglean013966.1
mdm-miR166g	Gglean013488.1
novel_mir511	Gglean029051.1
novel_mir469	Gglean016708.1
mdm-miR396c	Gglean029554.1
mdm-miR396c	Gglean000122.1
mdm-miR396c	Gglean030172.1
mdm-miR396c	Gglean011692.1
mdm-miR396c	Gglean017067.1
mdm-miR396c	Gglean002260.1

mdm-miR396c	Gglean012701.1
mdm-miR396c	Gglean026508.1
mdm-miR396c	Gglean029171.1
mdm-miR396c	Gglean015340.1
mdm-miR396c	Gglean006981.1
mdm-miR396c	Gglean013328.1
mdm-miR396c	Gglean024774.1
mdm-miR396c	Gglean012175.1
mdm-miR396c	Gglean023132.1
mdm-miR396c	Gglean002322.1
mdm-miR396c	Gglean025329.1
mdm-miR396c	Gglean028260.1
novel_mir375	Gglean007341.1
mdm-miR164f	Gglean022642.1
mdm-miR164f	Gglean005008.1
mdm-miR164f	Gglean003381.1
mdm-miR164f	Gglean001478.1
mdm-miR164f	Gglean007834.1
mdm-miR164f	Gglean007434.1
mdm-miR164f	Gglean016084.1
mdm-miR164f	Gglean025995.1
mdm-miR164f	Gglean000139.1
mdm-miR164f	Gglean027714.1
mdm-miR164f	Gglean031067.1
mdm-miR164f	Gglean026964.1
novel_mir349	Gglean028955.1
novel_mir349	Gglean021878.1
novel_mir349	Gglean015900.1
novel_mir349	Gglean004065.1
novel_mir349	Gglean011676.1
novel_mir349	Gglean011968.1
novel_mir349	Gglean000062.1
novel_mir349	Gglean026343.1
novel_mir349	Gglean028957.1
novel_mir349	Gglean015985.1
novel_mir349	Gglean026592.1
novel_mir349	Gglean007198.1
novel_mir349	Gglean024878.1
novel_mir349	Gglean016853.1
novel_mir349	Gglean004527.1
novel_mir349	Gglean006931.1
novel_mir349	Gglean018594.1
novel_mir349	Gglean028544.1
novel_mir349	Gglean026695.1

novel_mir349	Gglean001451.1
novel_mir349	Gglean008644.1
novel_mir349	Gglean031176.1
mdm-miR156k	Gglean028488.1
mdm-miR156k	Gglean028306.1
mdm-miR156k	Gglean004741.1
mdm-miR156k	Gglean029526.1
mdm-miR156k	Gglean005913.1
mdm-miR156k	Gglean005740.1
mdm-miR156k	Gglean016490.1
mdm-miR156k	Gglean009481.1
mdm-miR156k	Gglean016197.1
mdm-miR156k	Gglean000364.1
mdm-miR156k	Gglean005554.1
mdm-miR156k	Gglean000847.1
mdm-miR156k	Gglean007788.1
mdm-miR156k	Gglean001912.1
mdm-miR156k	Gglean008959.1
mdm-miR156k	Gglean017894.1
mdm-miR156k	Gglean016208.1
novel_mir177	Gglean009802.1
mdm-miR395d	Gglean006692.1
mdm-miR395d	Gglean005238.1
mdm-miR395d	Gglean030038.1
mdm-miR395d	Gglean022778.1
mdm-miR395d	Gglean015303.1
mdm-miR395d	Gglean008746.1

Supplementary Table S8 Primers of miRNA and their targets used in this study.

miRNA		
Primer ID	Stem loop reverse transcriptase primers	Forward primers
mdm-miR172b-RT	GTCGTATCCAGTGCAGGGTCCGAGGTATTCGCACTGGATACGACCGACGT	GCGGCGAGAATCTTGATGAT
mdm-miR164e-RT	GTCGTATCCAGTGCAGGGTCCGAGGTATTCGCACTGGATACGACGCACGT	GCGGCGTGGAGAAGCAGGGCA
mdm-miR396c-RT	GTCGTATCCAGTGCAGGGTCCGAGGTATTCGCACTGGATACGACCTTGAA	GCGGCGTTCCACAGCTTTCTT
mdm-miR164d-RT	GTCGTATCCAGTGCAGGGTCCGAGGTATTCGCACTGGATACGACGCACGT	GCGGCGGGGAGAAGCAGGGCA
novel_mir2-RT	GTCGTATCCAGTGCAGGGTCCGAGGTATTCGCACTGGATACGACTCCGCG	GCGGCGGATGGAATCGAGATCACAGA
novel_mir367-RT	GTCGTATCCAGTGCAGGGTCCGAGGTATTCGCACTGGATACGACGTAGGC	GCGGCGAGTTGCGCCCGAAGC
novel_mir3	GTCGTATCCAGTGCAGGGTCCGAGGTATTCG	GCGGCGTCTGAGCCTT

49-RT	CACTGGATACGACGGGCGA	CGG
Primer ID	Universal reverse primer	
	GTGCAGGGTCCGAGGT	
miRNA-target		
Primer ID	Forward primers (5'to3')	Reverse primer (5'to3')
Gglean031260.1	TGGCAAGTGAATGGCTCAGT	CTGACCCGGAAGGCGAATAA
Gglean008425.1	TGCATTTCAGACGAGGAGCC	GATTTCTCGACGGGGTGAGT
Gglean027146.1	CAGTACTCCACCACGACCAC	GCAGCTGCAATCTCGTTAGC
Gglean029881.1	GGGCTTTCGGGTCAATACCA	TGCAATTTCTGTGTCGCGTG
Gglean026000.1	CCATGCTGGACTGGGTAAGG	CCGACATCTTAGGCCTGTGG
Gglean023388.1	TGCGAATCCCACCATCAACA	GATGGCACCGACCATAGAGG
Gglean003381.1	CCTAAACGCAGTGCCTACCA	ATGCAGGAACCACCTAAGCC
Gglean029554.1	ACGGGGTGGAACAAGATCAC	ACAGTCCAAGCAGCCAAAGA
Gglean005008.1	TGATGCGATCGAGCTTGAGG	AAGCATCCGTACCACTTCCG
Gglean026964.1	TAGGCTCCGGTATGAACCCA	CACCCGAGCAACTTCTTTGC
Gglean021878.1	GGTCTCACCAGCGATCCAAA	TAGTGTGAGCCTCGTGCTTG
